# Versatility or Promiscuity: The Estrogen Receptors, Control of Ligand Selectivity and an Update on Subtype Selective Ligands

**DOI:** 10.3390/ijerph110908709

**Published:** 2014-08-26

**Authors:** Hui Wen Ng, Roger Perkins, Weida Tong, Huixiao Hong

**Affiliations:** Division of Bioinformatics and Biostatistics, National Center for Toxicological Research, US Food and Drug Administration, 3900 NCTR Road, Jefferson, AR 72079, USA; E-Mails: Huiwen.Ng@fda.hhs.gov (H.W.N.); Roger.Perkins@fda.hhs.gov (R.P.); Weida.Tong@fda.hhs.gov (W.T.)

**Keywords:** estrogen receptors (ERs), estrogen receptor alpha (ERα), estrogen receptor beta (ERβ), promiscuity, ligand selectivity, subtype selective ligands

## Abstract

The estrogen receptors (ERs) are a group of versatile receptors. They regulate an enormity of processes starting in early life and continuing through sexual reproduction, development, and end of life. This review provides a background and structural perspective for the ERs as part of the nuclear receptor superfamily and discusses the ER versatility and promiscuity. The wide repertoire of ER actions is mediated mostly through ligand-activated transcription factors and many DNA response elements in most tissues and organs. Their versatility, however, comes with the drawback of promiscuous interactions with structurally diverse exogenous chemicals with potential for a wide range of adverse health outcomes. Even when interacting with endogenous hormones, ER actions can have adverse effects in disease progression. Finally, how nature controls ER specificity and how the subtle differences in receptor subtypes are exploited in pharmaceutical design to achieve binding specificity and subtype selectivity for desired biological response are discussed. The intent of this review is to complement the large body of literature with emphasis on most recent developments in selective ER ligands.

## 1. The Estrogen Receptors

**Figure 1:**
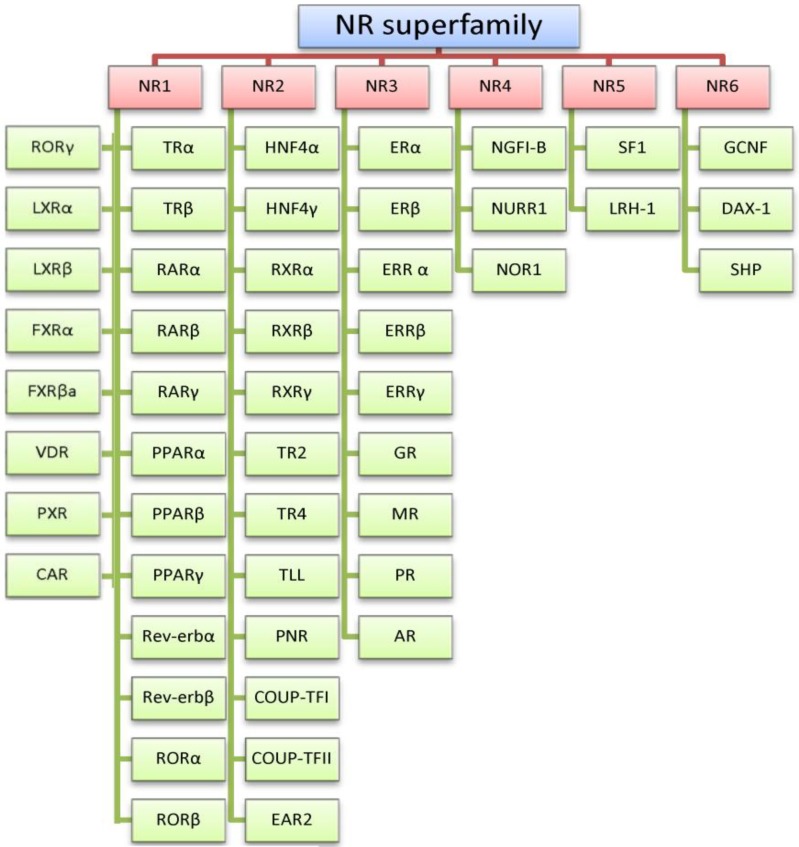
Taxonomy of the nuclear receptor (NR) superfamily and members of the families 1-6 (NR1-6).

The estrogen receptors (ERs) are members of the steroid hormone receptor family, which taxonomically are within the nuclear receptor (NR) superfamily (Figure 1). The steroid hormone receptor family (a.k.a. NR3) has many members, including the mineralocorticoid, glucocorticoid, progesterone, androgen, and estrogen-related receptors (MRs, GRs, PRs, ARs and ERRs, respectively) [1,2]. These receptors are targets of the lipophilic steroid hormones sharing cholesterol as a common building block and are synthesized in the adrenal cortex (e.g., glucocorticoids, mineralocorticoids and adrenal androgens), testes (e.g., testosterone and testicular androgens), ovary and placenta (estrogens and progesterone) [3].

The role of the ERs far transcends the most commonly ascribed purposes of regulating development of female characteristics and the female reproductive system. ERs are also indispensable in a diversity of physiological processes that include regulation of lipid profile, bone integrity, hemostasis, endothelial functions, inflammatory markers, the growth of different tissues, and both prenatal and postnatal development [4,5,6]. This extensive involvement may be attributable to the status of an ER as the common ancestor of members of the steroid hormone receptor family [1,7]. Estrogen mediates both male and female health, having both adverse and beneficial effects in both sexes. For example, the ERs and their hormone ligands are implicated in stroke [8], cancers (e.g., endometrial [7] and breast [9]), as well as postmenopausal symptoms resulting from depleted circulating estrogen (e.g., osteoporosis, hot flushes and vaginal atrophy).

**Figure 2:**
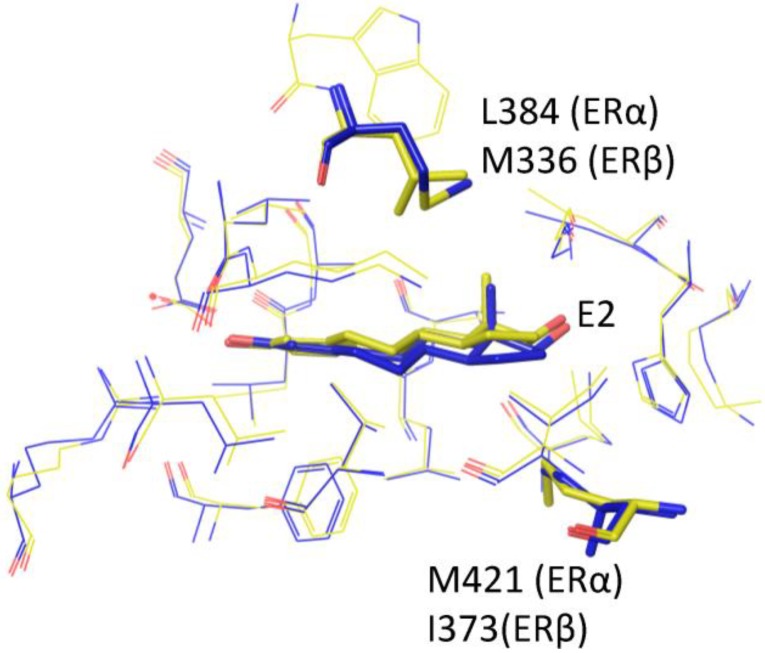
17β-estradiol (E2) bound to ERα (yellow) and ERβ (blue). Only two residues, *i.e.*, L384/M336 and M421/I373 (Erα/ERβ), differ in the binding pockets of ERα and ERβ. Unsurprisingly, the E2 binds in the subtypes in only subtlely different manners.

The ERs are divided into two major subtypes, ERα and ERβ, which possess distinctly different identities [5,10,11]. A third subtype, ERγ, found only in non-human species e.g., fish, has also been reported [12]. The ERα and ERβ subtypes are coded by different genes, *ESR1* and *ESR2* respectively, and are distributed and play multiple roles in a tissue-dependent manner. For example, the ERα is more prevalent in the gonads, mammary glands, kidney and lung bronchi, while ERβ predominates in bone, lung alveoli and prostate tissues [5,13,14]; furthermore, ERα has been found to promote cell proliferation while ERβ possesses an anti-proliferative effect in the mammary tissues [15,16,17]. The ERα and ERβ appear to share a modest 47% [10,18] and 56% [5], respectively, overall and ligand binding domain (LBD) sequence identity; yet, only two residues among those that line the binding pocket are found to be different: Leu384 (ERα) *vs.* Met336 (ERβ) and Met421(ERα) *vs.* Ile373 (ERβ) [5] (Figure 2).

NR1 consists of TR: thyroid receptor; RAR: retinoic acid receptor; PPAR: peroxisome proliferator-activated receptor; ROR: retinoic acid related orphan nuclear receptor; LXR: liver X receptor; FXR: farnesoid X receptor; VDR: vitamin D receptor; PXR: pregnane X receptor and CAR: constitutive androstane receptor. Note: no full terminology for Rev-erb. NR2 consists of HNF: hepatocyte nuclear factor; RXR: retinoid X receptor; TR: testicular receptor; TLL: tailless-like receptor; PNR: photoreceptor-specific nuclear receptor; COUP-TF: chicken ovalbumin upstream promoter transcription factor and EAR: ErbA-related protein. NR 3 consists of ER: estrogen receptor; ERR: estrogen-related receptor; GR: glucocorticoid receptor; MR: mineralocorticoid receptor; PR: progesterone receptor and AR: androgen receptor. NR4 consists of NGFI-B: nerve growth factor-induced clone B; NURR: Nur-related factor and NOR: neuron-derived orphan receptor. NR5 consists of SF1: steroidogenic factor 1 and LRH: liver receptor homolog. NR6 consists of GCNF: germ cell nuclear factor; DAX-1: dosage-sensitive sex reversal-adrenal hypoplasia congenital critical region on the X chromosome protein 1 and SHP: small heterodimer partner. Table adapted from [19,20].

## 2. Estrogen Receptor Architecture

**Figure 3:**
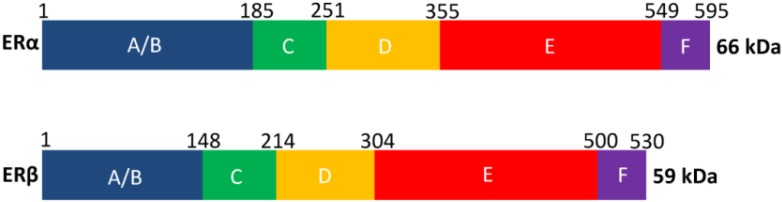
Domains A-F of ERα and ERβ, each playing a different structural/functional role. The numbers above the bars denote the residue numbers in the two receptor subtypes. ERα is slightly larger than ERβ, with a total of 595 amino acids (66 kDa) compared to 530 amino acids (59 kDa) for ERβ.

The ERs share a common architecture with other members of the NR superfamily that are composed of six evolutionarily conserved domains A, B, C, D, E and F (Figure 3). The solvent-exposed A/B region located at the N-terminus contains the ligand-independent activation function 1 (AF1) and is responsible for protein-protein interactions [2,5]. The highly conserved C region is the DNA binding domain, and is responsible for DNA dependent and independent receptor dimerization [21], as well as for binding to specific regions of the DNA (e.g., the estrogen response elements, EREs) [5]. The little-known and poorly conserved D region is the linker region that contains the nuclear localization sequence as well as sites for co-modulator recruitment and post-translational modifications. The E and F regions located at the C terminus contain the LBD and the ligand-dependent activation function 2 domain (AF2), as well as sections for nuclear localization and homo/hetero dimerization [5,22]. It is worth noting that several splice variants exist for both ERα (over twenty) and ERβ (at least five) subtypes [23,24,25]. While the wild-type ERα and ERβ (66 and 59kDa, respectively) contain complete A-F regions and are considered as full-length, the alternative splice variants either lack a portion of the protein or contain duplicate regions compared to the wild-type ERs [23]. Examples of splice variants lacking a portion of the protein are the ERα36 and ERβ2 [2,23]. The former, devoid of the AF1 region, has been found to oppose ERα AF1-dependent transcription [26]; the latter, shorter around the AF2 region due to shorter helix 11, has an altered AF2 conformation that results in limited ligand access [27]. The ERα80 isoform is an example of a splice variant which contains duplicate region as demonstrated by its extended E domain; so far, its function remains unclear [28]. More comprehensive discussions of the ER splice variants are provided by Sotoca *et al.* [23], Lewandowski *et al.* [25] and Poola *et al.* [24].

The LBDs of the ERs have been of intense interest to the scientific community and pharmaceutical industry for obvious reasons: the conformational changes that occur in the LBD following ligand and co-regulator binding determine and often initiate a spectrum of important and different downstream estrogen responses. Concomitantly, the binding site is the target for designing ligands causing a specific and large response of a certain type, called subtype selectivity. This is also precisely the reason why a great number of crystal structures of ERα and ERβ LBDs in complex with a multitude of ligands have been elucidated over the years (See Table 1 and Table 2 for lists of ERα and ERβ LBD crystal structures available to date in the Protein Data Bank (PDB)).

**Table 1:** Crystal structures of ERα ligand binding domain bound with ligands in PDB.

PDB ID	Structure-Type	Ligand	Res.(Å)	Ref
1A52	Dimer	Estradiol	2.8	[29]
1ERE	Hexamer	Estradiol	3.1	[30]
1ERR	Dimer	Raloxifene	2.6	[30]
3ERD	Dimer	Diethylstilbestrol	2.03	[31]
3ERT	Monomer	4-Hydroxytamoxifen	1.9	[31]
1QKT	Monomer	Estradiol	2.2	[32]
1QKU	Trimer	Estradiol	3.2	[32]
1G50	Trimer	Estradiol	2.9	[33]
1GWQ	Dimer	Raloxifene core	2.45	[34]
1GWR	Dimer	Estradiol	2.4	[34]
1L2I	Dimer	(*R,R*)-5,11-*cis*-Diethyl-5,6,11,12-tetrahydrochrysene- 2,8-diol	1.95	[35]
1PCG	Dimer	Estradiol	2.7	[36]
1UOM	Monomer	2-Phenyl-1-[4-(2-piperidin-1-yl-ethoxy)-phenyl]-1,2,3, 4-tetrahydroisoquinolin-6-ol	2.28	[37]
1R5K	Trimer	(2*E*)-3-{4-[(1*E*)-1,2-Diphenylbut-1-enyl]phenyl}acrylic acid	2.7	[38]
1SJ0	Monomer	(2*S*,3*R*)-2-(4-(2-(Piperidin-1-yl)ethoxy)phenyl)-2,3-dihydro-3-(4-hydroxyphenyl)benzo[b][1,4]oxathiin-6-ol	1.9	[39]
1XP1	Monomer	(2*S*,3*R*)-2-(4-{2-[(3*R*,4*R*)-3,4-Dimethylpyrrolidin-1-yl]ethoxy}phenyl)-3-(4-hydroxyphenyl)-2,3-dihydro-1,4-benzoxathiin-6-ol	1.8	[40]
1XP6	Monomer	(2*S*,3*R*)-2-(4-{2-[(3*S*,4*S*)-3,4-Dimethylpyrrolidin-1-yl]ethoxy}phenyl)-3-(4-hydroxyphenyl)-2,3-dihydro-1,4-benzoxathiin-6-ol	1.7	[40]
1XP9	Monomer	(2*S*,3*R*)-3-(4-Hydroxyphenyl)-2-(4-{[(2*S*)-2-pyrrolidin-1-ylpropyl]oxy}phenyl)-2,3-dihydro-1,4-benzoxathiin-6-ol	1.8	[40]
1XPC	Monomer	(2*S*,3*R*)-3-(4-Hydroxyphenyl)-2-(4-{[(2*R*)-2-pyrrolidin-1-ylpropyl]oxy}phenyl)-2,3-dihydro-1,4-benzoxathiin-6-ol	1.6	[40]
1X7E	Dimer	[5-Hydroxy-2-(4-hydroxyphenyl)-1-benzofuran-7-yl]acetonitrile	2.8	[41]
1X7R	Monomer	Genistein	2	[42]
1XQC	Tetramer	(1*S*)-1-{4-[(9a*R*)-Octahydro-2*H*-pyrido[1,2-a]pyrazin-2-yl]phenyl}-2-phenyl-1,2,3,4-tetrahydroisoquinolin-6-ol	2.05	[43]
1YIM	Monomer	(2*R*,3*R*,4*S*)-3-(4-Hydroxyphenyl)-4-methyl-2-[4-(2-pyrrolidin-1-ylethoxy)phenyl]chroman-6-ol	1.9	[44]
1YIN	Monomer	(2*R*,3*R*,4*S*)-5-Fluoro-3-(4-hydroxyphenyl)-4-methyl-2-[4-(2-piperidin-1-ylethoxy)phenyl]chroman-6-ol	2.2	[44]
2AYR	Monomer	6-(4-Methylsulfonyl-phenyl)-5-[4-(2-piperidin-1-ylethoxy)phenoxy]naphthalen-2-ol	1.9	[45]
2B23	Dimer		2.1	[46]
2BJ4	Dimer	4-Hydroxytamoxifen	2	[47]
1ZKY	Dimer	4-[(1*S*,2*S*,5*S*)-5-(Hydroxymethyl)-6,8,9-trimethyl-3-oxabicyclo[3.3.1]non-7-en-2-yl]phenol	2.25	[48]
2B1V	Dimer	4-[(1*S*,2*S*,5*S*)-5-(Hydroxymethyl)-8-methyl-3-oxabicyclo[3.3.1]non-7-en-2-yl]phenol	1.8	[48]
2B1Z	Dimer	17-Methyl-17-α-dihydroequilenin	1.78	[49]
2FAI	Dimer	4-[(1*S*,2*S*,5*S*,9*R*)-5-(Hydroxymethyl)-8,9-dimethyl-3-oxabicyclo[3.3.1]non-7-en-2-yl]phenol	2.1	[48]
2I0J	Tetramer	(3a*S*,4*R*,9b*R*)-4-(4-Hydroxyphenyl)-1,2,3,3a,4,9b-hexahydrocyclopenta[c]chromen-8-ol	2.9	[50]
2G44	Dimer	4-[(1*S*,2*R*,5*S*)-4,4,8-Trimethyl-3-oxabicyclo[3.3.1]non-7-en-2-yl]phenol	2.65	-
2G5O	Dimer	(9α,13β,17β)-2-[(1*Z*)-But-1-en-1-yl]estra-1,3,5(10)-triene-3,17-diol	2.3	-
2IOG	Monomer	N-[(1*R*)-3-(4-Hydroxyphenyl)-1-methylpropyl]-2-[2-phenyl-6-(2-piperidin-1-ylethoxy)-1h-indol-3-yl]acetamide	1.6	[51]
2IOK	Dimer	N-[(1*R*)-3-(4-Hydroxyphenyl)-1-methylpropyl]-2-(2-phenyl-1*H*-indol-3-yl)acetamide	2.4	[51]
2JF9	Trimer	4-Hydroxytamoxifen	2.1	[52]
2JFA	Dimer	Raloxifene	2.55	[52]
2OCF	Monomer	Estradiol	2.95	[53]
2OUZ	Monomer	(5*R*,6*S*)-6-Phenyl-5-[4-(2-pyrrolidin-1-ylethoxy)phenyl]-5,6,7,8-tetrahydronaphthalen-2-ol	2	[54]
2P15	Dimer	(17β)-17-{(*E*)-2-[2-(Trifluoromethyl)phenyl]vinyl}estra-1(10),2,4-triene-3,17-diol	1.94	[55]
2POG	Dimer	(3a*S*,4*R*,9b*R*)-4-(4-Hydroxyphenyl)-1,2,3,3a,4,9b-hexahydrocyclopenta[c]chromen-9-ol	1.84	[56]
2Q6J	Dimer	4-[(Dimesitylboryl)(2,2,2-trifluoroethyl)amino]phenol	2.7	[57]
2Q70	Dimer	(3a*S*,4*R*,9b*R*)-2,2-Difluoro-4-(4-hydroxyphenyl)-1,2,3,3a,4,9b-hexahydrocyclopenta[c]chromen-8-ol	1.95	[58]
2QE4	Dimer	(3a*S*,4*R*,9b*R*)-4-(4-Hydroxyphenyl)-6-(methoxymethyl)-1,2,3,3a,4,9b-hexahydrocyclopenta[c]chromen-8-ol	2.4	[50]
2QA6	Dimer	4-(6-Hydroxy-1*H*-indazol-3-yl)benzene-1,3-diol	2.6	[46]
2QA8	Dimer	Genistein	1.85	[46]
2QAB	Dimer	3-Ethyl-2-(4-hydroxyphenyl)-2*H*-indazol-5- ol	1.89	[46]
2QGT	Dimer	(9β,11α,13α,14β,17α)-11-(methoxymethyl)estra-1(10),2,4-triene-3,17-diol	2.15	[46]
2QGW	Dimer	3-Chloro-2-(4-hydroxyphenyl)-2*H*-indazol-5-ol	2.39	[46]
2QH6	Dimer	Diethyl (1*R*,2*S*,3*R*,4*S*)-5,6-bis(4-hydroxyphenyl)-7-oxabicyclo[2.2.1]hept-5-ene-2,3-dicarboxylate	2.7	[46]
2QR9	Dimer	Dimethyl (1*R*,4*S*)-5,6-bis(4-hydroxyphenyl)-7-oxabicyclo[2.2.1]hepta-2,5-diene-2,3-dicarboxylate	2	[46]
2QSE	Dimer	4-(2-Amino-1-methyl-1*H*-imidazo[4,5-b]pyridin-6-yl)phenol	1.85	[46]
2QXM	Dimer	2-Amino-1-methyl-6-phenylimidazo[4,5-b]pyrid	2.3	[46]
2QXS	Dimer	Raloxifene	1.7	[59]
2QZO	Dimer	4-[1-Allyl-7-(trifluoromethyl)-1h-indazol- 3-yl]bezene-1,3-diol	1.72	[59]
2R6W	Dimer	[6-Hydroxy-2-(4-hydroxyphenyl)-1-benzothien-3-yl]{4-[2-(4-methylpiperidin-1-yl)ethoxy]phenyl}methanone	2	[60]
2R6Y	Dimer	[6-Hydroxy-2-(4-hydroxyphenyl)-1-benzothien-3-yl][4-(2-pyrrolidin-1-ylethoxy)phenyl]methanone	2	[60]
3DT3	Dimer	5-(4-Hydroxyphenoxy)-6-(3-hydroxyphenyl)- 7-methylnapthalen-2-ol	2.4	[61]
3HLV	Dimer	(9β,13α,16β)-3,16-Dihydroxyestra- 1,3,5(10)-trien-17-one	3	-
3HM1	Dimer	(9β,13α)-3-Hydroxyestra-1,3,5(10)-trien-17-one	2.33	-
3L03	Dimer	(14β,15α,16α,17α)-Estra-1,3,5(10)-triene-3,15,16,17-tetrol	1.9	-
3OS8	Tetramer	4-[1-Benzyl-7-(trifluoromethyl)-1*H*-indazol-3-yl]benzene-1, 3-diol	2.03	[59]
3OS9	Tetramer	4-[1-Allyl-7-(trifluoromethyl)-1*H*-indazol-3-yl]benzene-1,3-diol	2.3	[59]
3OSA	Tetramer	4-[1-(3-Methylbut-2-en-1-yl)-7-(trifluoromethyl)-1*H*-indazol-3-yl]benzene-1,3-diol	2.3	[59]
2YAT	Monomer	Estradiol-pyridinium tetraacetic acid	2.6	[62]
2YJA	Monomer	Estradiol	1.82	[63]
3Q95	Dimer	Estriol	2.05	-
3Q97	Dimer	4,4’-[(1*Z*)-1-(4-Ethoxyphenyl)but-1-ene-1,2-diyl]diphenol; 4,4’-[2-(4-Ethoxyphenyl)but-1-ene-1,1-diyl]diphenol	2.1	-
3UU7	Dimer	4,4’-Propane-2,2-diyldiphenol	2.2	[64]
3UUA	Dimer	4,4’-(1,1,1,3,3,3-Hexafluoropropane-2,2-diyl)diphenol	2.05	[64]
3UUC	Tetramer	4,4’-(2,2-Dichloroethene-1,1-diyl)diphenol	2.1	[64]
3UUD	Dimer	Estradiol	1.6	[64]
4DMA	Dimer	2’-Bromo-6’-(furan-3-yl)-4’-(hydroxymethyl)biphenyl-4-ol	2.3	[65]
4IU7	Dimer	4-[2-Ethyl-7-(trifluoromethyl)-2*H*-indazol-3-yl]benzene-1, 3-diol	2.29	[66]
4IUI	Dimer	4-[1-Butyl-7-(trifluoromethyl)-1*H*-indazol-3-yl]benzene-1, 3-diol	2.3	[66]
4IV2	Dimer	4-[1-(2-Methylpropyl)-7-(trifluoromethyl)-1*H*-indazol-3-yl]benzene-1,3-diol	2.14	[66]
4IV4	Dimer	4-[2-(2-Methylpropyl)-7-(trifluoromethyl)- 2h-indazol-3-yl]benzene-1,3-diol	2.3	[66]
4IVW	Dimer	4-[2-Benzyl-7-(trifluoromethyl)-2*H*-indazol-3-yl]benzene-1,3-diol	2.06	[66]
4IVY	Dimer	4-[1-(But-3-en-1-yl)-7-(trifluoromethyl)-1*H*-indazol-3-yl]benzene-1,3-diol	1.95	[66]
4IW6	Dimer	4-[2-(But-3-en-1-yl)-7-(trifluoromethyl)-2*H*-indazol-3-yl]benzene-1,3-diol	1.98	[66]
4IW8	Dimer	4-[1-(3-Methylbut-2-en-1-yl)-7-(trifluoromethyl)-1*H*-indazol-3-yl]benzene-1,3-diol	2.04	[66]
4IWC	Dimer	4,4’-Thiene-2,5-diylbis(3-methylphenol)	2.24	[66]
4IWF	Dimer	2-Chloro-3’-fluoro-3-[(*E*)-(hydroxyimino)methyl]biphenyl- 4,4’-diol	1.93	[66]

**Table 2:** Crystal structures of ERβ ligand binding domain bound with ligands in PDB.

PDB ID	Structure-Type	Ligand	Res. (Å)	Ref
1QKM	Monomer	Genistein	1.8	[67]
1QKN	Monomer	Raloxifene	2.25	[67]
1HJ1	Monomer	ICI164384 or N-Butyl-11-[(7r,8r,9s,13s,14s,17s)-3,17-dihydroxy-13-methyl-7,8,9,11,12,13,14,15,16,17- decahydro- 6 *H*-cyclopenta[a]phenanthren-7-yl]-*n*-methylundecanamide	2.3	[68]
1L2J	Dimer	(*R*,*R*)-5,11-*cis*-Diethyl-5,6,11,12-tetrahydrochrysene-2,8-diol	2.95	[35]
1NDE	Monomer	4-(2-{[4-{[3-(4-Chlorophenyl)propyl]sulfanyl}-6-(1-piperazinyl)-1,3,5-triazin-2-yl]amino}ethyl)phenol	3	[69]
1U3Q	Tetramer	4-(6-Hydroxybenzo[d]isoxazol-3-yl)benzene-1,3-diol	2.4	[70]
1U3R	Dimer	2-(5-Hydroxynaphthalen-1-yl)-1,3-benzooxazol-6-ol	2.21	[70]
1U3S	Dimer	3-(6-Hydroxynaphthalen-2-yl)-benzo[d]isooxazol-6-ol	2.5	[70]
1U9E	Dimer	2-(4-Hydroxyphenyl)benzofuran-5-ol	2.4	[41]
1X76	Dimer	5-Hydroxy-2-(4-hydroxyphenyl)-1-benzofuran-7-carbonitrile	2.2	[41]
1X78	Dimer	[5-Hydroxy-2-(4-hydroxyphenyl)-1-benzofuran-7-yl]acetonitrile	2.3	[41]
1X7B	Dimer	2-(3-Fluoro-4-hydroxyphenyl)-7-vinyl-1,3-benzoxazol-5-ol	2.3	[41]
1X7J	Dimer	Genistein	2.3	[41]
1YY4	Dimer	1-Chloro-6-(4-hydroxyphenyl)-2-naphthol	2.7	[71]
1YYE	Dimer	3-(3-Fluoro-4-hydroxyphenyl)-7-hydroxy-1-naphthonitrile	2.03	[71]
1ZAF	Dimer	3-Bromo-6-hydroxy-2-(4-hydroxyphenyl)-1*H*-inden-1-one	2.2	[72]
2FSZ	Dimer	4-Hydroxytamoxifen	2.2	[73]
2GIU	Monomer	(9a*S*)-4-bromo-9a-butyl-7-hydroxy-1,2,9,9a-tetrahydro-3*H*-fluoren-3-one	2.2	[74]
2I0G	Dimer	(3a*S*,4*R*,9b*R*)-4-(4-hydroxyphenyl)-1,2,3,3a,4,9b-hexahydrocyclopenta[c]chromen-8-ol	2.5	[75]
2J7X	Monomer	Estradiol	2.1	-
2J7Y	Monomer	(16α,17α)-Estra-1,3,5(10)-triene- 3,16,17-triol	1.8	-
2JJ3	Dimer	(3a*S*,4*R*,9b*R*)-4-(4-Hydroxyphenyl)-6-(methoxymethyl)-1,2,3,3a,4,9b-hexahydrocyclopenta[c]chromen-8-ol	2.28	[50]
2NV7	Dimer	4-(4-Hydroxyphenyl)-1-naphthaldehyde oxime	2.1	[76]
2QTU	Dimer	(3a*S*,4*R*,9b*R*)-2,2-Difluoro-4-(4-hydroxyphenyl)-6-(methoxymethyl)-1,2,3,3a,4,9b-hexahydrocyclopental[c]chromen-8-ol	2.53	[77]
2Z4B	Dimer	(3a*S*,4*R*,9b*R*)-2,2-Difluoro-4-(4-hydroxyphenyl)-1,2,3,3a,4,9b-hexahydrocyclopenta[c]chromen-8-ol	2.34	[58]
3OLL	Dimer	Estradiol	1.5	[78]
2YJD	Dimer	4-(2-Propan-2-yloxybenzimidazol-1-yl)phenol	1.93	[63]
3OLS	Dimer	Estradiol	2.2	[78]
3OMO	Dimer	2-(Trifluoroacetyl)-1,2,3,4-tetrahydroisoquinolin-6-ol	2.21	[79]
3OMP	Dimer	2-(Trifluoroacetyl)-1,2,3,4-tetrahydroisoquinolin-7-ol	2.05	[79]
3OMQ	Dimer	2-[(Trifluoromethyl)sulfonyl]-1,2,3,4-tetrahydroisoquinolin-6-ol	1.97	[79]
2YLY	Dimer	N-Cyclopropyl-4-oxidanyl-N-[(2*R*)-2-oxidanyl-2-phenylpropyl]benzenesulfonamide	3.2	[80]
4J24	Tetramer	Estradiol	2.1	[81]
4J26	Dimer	Estradiol	2.3	[81]

**Figure 4:**
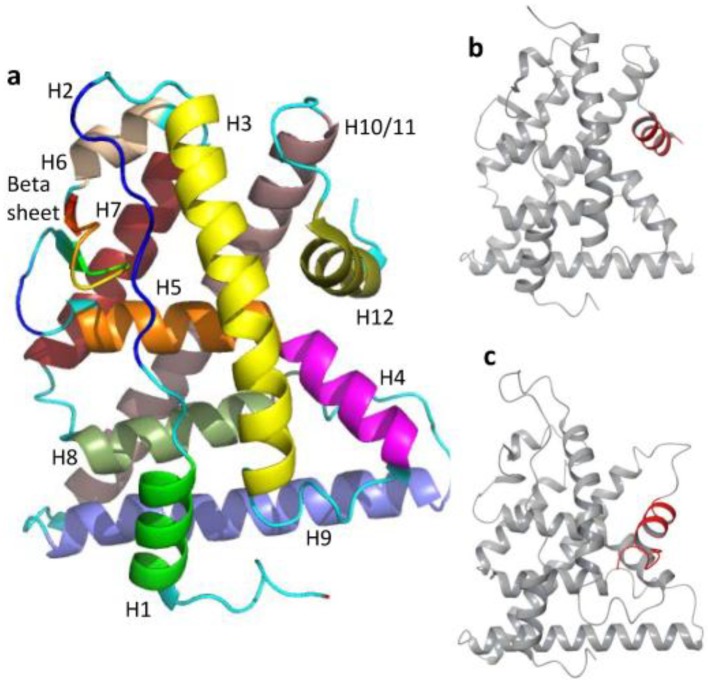
General architecture of an ER LBD comprising twelve α-helices and a beta sheet/hairpin. The twelve α-helices (H1 to H12) that form the “three-layered anti-parallel α helical sandwich” are colored differently for clarity (**a**). The conformation of an active ER (PDB ID: 1GWR) (**b**). The conformation of an inactive ER (PDB ID: 3ERT) (**c**). The major difference between **b** and **c** lies in the H12 conformation, highlighted in red.

Commonly described as a “three-layered anti-parallel α helical sandwich”, the LBD consists of 12 helices (H1 to H12) and a beta hairpin, with a core region made up of H5, H6, H9 and H10, as well as two flanking outer layers comprising the remaining helices [5,30] (Figure 4a). The H12 that sits at the end of the LBD forms the AF2 with H3, H4 and H5, and plays a key role as a molecular switch [55]. Influenced by ligand binding (although without direct ligand contact [29]), H12 regulates receptor activities and the recruitment of co-regulators to AF2 by adopting distinct conformations, *i.e.*, active *vs.* inactive conformations [82] (Figure 4b,c, respectively). In the active conformation, H12 rests across H3 and H11, forming a groove to accommodate co-regulator binding; in the inactive conformation, such as when bound to an antagonist, H12 is displaced from this position, which distorts the co-regulator binding groove [59]. Flanked by the beta hairpin and H12, the ligand binding cavity (made up of H3, H4, H10 and H11) is a compact and enclosed ellipsoid cavity situated deep in the core of the LBD [29,82].

## 3. Promiscuity of Estrogen Receptors

**Figure 5:**
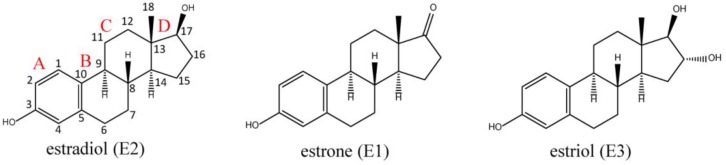
Structures of natural estrogens: estradiol (E2, the most potent), estrone (E1) and estriol (E3). The four rings of the endogenous ligand, E2, are labelled A-D according to the widely accepted naming convention.

The ERs are the target of the natural estrogens, namely estradiol (E2, most potent) [83], estrone (E1) and estriol (E3). E2 is also commonly referred to as 17β-estradiol as it possesses the hydroxyl group at the carbon 17 position above the steroid plane (α and β indicate below and above the steroid plane, respectively) (Figure 5).

**Figure 6:**
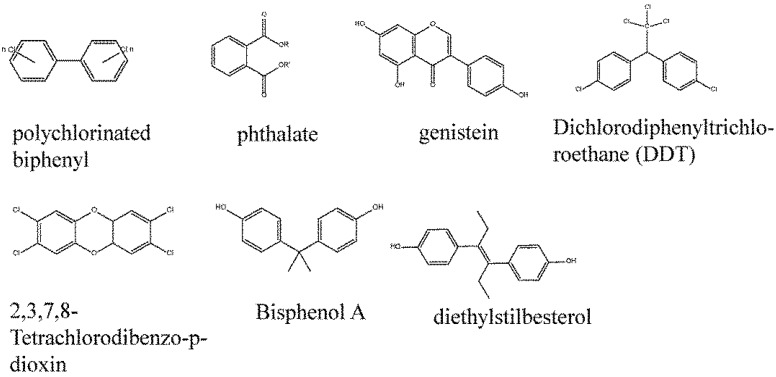
The diverse structures of ER-binding ligands. Most notably, these ligands contain at least an aromatic ring, a feature believed to confer the ability to bind the ERs.

Apart from these endogenous ligands, the ERs, quite unlike the other members of the steroid receptor family, are also found to bind to a remarkably diverse range of exogenous substances [84,85,86], earning them a notorious reputation as the promiscuous receptors. Molecules that are able to bind to the ERs span from industrial byproducts (polychlorinated biphenyls, dioxins, e.g., 2,3,7,8-tetrachlorodibenzo-*p*-dioxin), plasticizer (phthalates), plastics (bisphenol A), naturally occurring phytoestrogens (genistein), pharmaceuticals products (diethylstilbesterol) to pesticides (dichlorodiphenyltrichloroethane, popularly known as DDT [87,88]). Despite the structural diversity (Figure 6), these molecules appear to possess at least an aromatic ring structure, which is believed to be crucial for having affinity to bind [84,89,90,91,92]. Now frequently referred to as endocrine disruptors, these compounds and a large number more have been implicated in numerous and diverse adverse health effects. Worldwide, authorities regulating drugs, foods, food packaging, veterinary products and medical devices now consider endocrine activity in their regulatory duties, as do authorities responsible for regulating chemicals in the environment [93]. Some examples of the adverse health outcomes include infertility, precocious puberty, various cancers (e.g., breast [94,95], cervical and vaginal cancers [96,97,98]), obesity, diabetes, cardiovascular [87,99] and immune disorders [100].

## 4. Root of ER Promiscuity

Ligand recognition and receptor-ligand binding are generally deemed as biological processes that take place with considerably high specificity; this is pivotal in allowing the body to “switch on” only the correct receptors to produce responses appropriate to various *in vivo*/environmental stimuli amidst the highly complex labyrinth of signaling pathways. The conventional “lock-and-key” model as well as the refined “induced fit” model has been widely accepted to underlie the modus operandi of protein-ligand recognition. These models suggest that the binding site of a receptor only recognizes specific ligands (or those that closely resemble them), bearing complementary size, shape and molecular properties, to the binding site. The fairly “rigid” rules that govern protein-ligand recognition extend further to the ligand binding modes whereby it is widely assumed that a ligand binds to the receptor binding site in a single conformation. This is understandably so as a great number of X-ray structures had supported this point.

**Figure 7:**
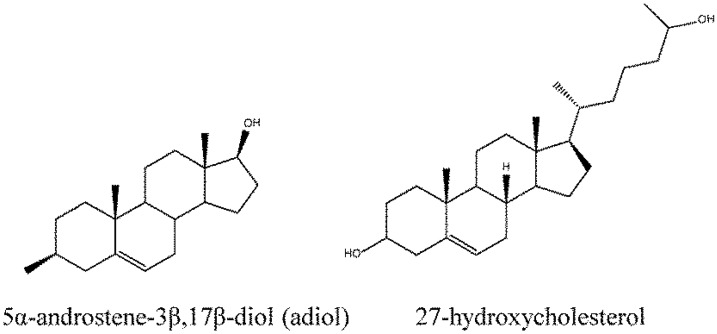
Other endogenous ligands of the ERs, 5α-androstene-3β,17β-diol (adiol) and 27-hydroxycholesterol (27OH).

The ERs have appeared to be an exception to many aspects of the core concept of lock and key that implies high specificity. In fact, ER ligands have been found to bind to the ER binding pocket in more than one binding orientation which, in turn, affect the final conformations (active/inactive) of the LBD [66]. The apparent lack of specificity, or rather, the high flexibility as demonstrated by the ER in ligand binding can perhaps be justified by the versatility and multi-functional roles of this group of receptors (see review by Lathe and Kotelevtsev [101]). Broadly speaking, the observed flexibility is required for: (1) binding to different endogenous ligands that vary according to tissue type, (2) multifunctional biological roles played by the ERs, and (3) graded activity *i.e.*, partial agonism and antagonism. For the first instance, while E2 is commonly seen as the default endogenous ligand for the ERs, this is not always the case: the 5α-androstene-3β,17β-diol (adiol) is found to replace the E2 as the physiological ligand for ERβ in prostate tissue, microglia and astrocytes; 27-hydroxycholesterol (27OHC) has been found to be the first endogenous selective estrogen receptor modulator (SERM) that acts as the modulator of both ERα and ERβ [101,102,103,104] (Figure 7). The existence of these additional endogenous ligands demonstrates the degree of binding flexibility required for ERs to fulfill their multiple functions. As for multifunctional biological roles of ERs, contrary to the oversimplified concept of a ligand (*i.e.*, an agonist or an antagonist) binding to the receptor with a specific conformation thus leading to a response (*i.e.*, activation or inhibition), the ERs paint a more multifaceted picture of receptor signaling. In the case of ERs, the binding of different ligands, depending on their nature and pharmacological classes, will lead to different LBD conformations and subsequent recruitment of co-regulator proteins. These different conformations and co-regulators, in turn, lead to different signaling through specific secondary pathways [46,82,101,105], eventually producing distinctly different biological responses. For graded activity, Bruning *et al*. [59] have shown that the ligand binding orientations in the binding pocket affect signaling outcomes which lead to a gradation of activities such as those expected of the partial agonists or antagonists.

**Figure 8:**
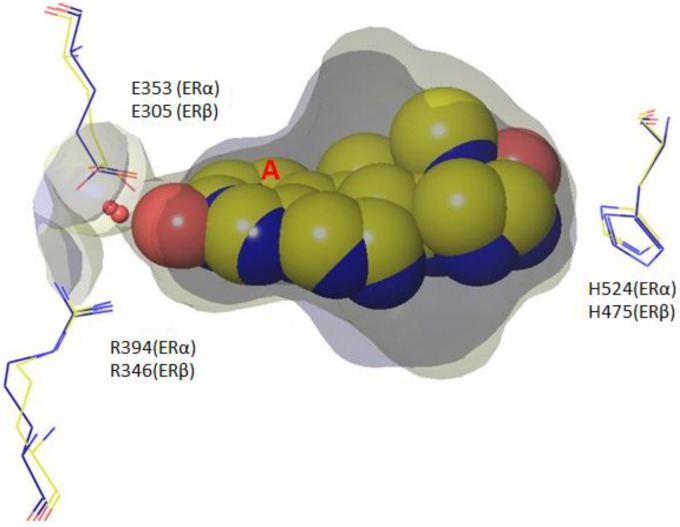
The binding of E2 in the pocket of ERα (PDB ID: 1GWR, yellow) and ERβ (PDB ID: 3OLL, blue), which are more or less comparable in size and volume. The phenolic A ring binds tightly to the pocket forming a triumvirate hydrogen bond network with a crystallographic water (red sphere), E353/305 (ERα/ERβ) and R294/246(ERα/ERβ) while the D ring forms a single hyrdrogen bond with H524/475 (ERα/ERβ). Apart from the A ring, the rest of the E2 molecule possesses high conformational flexibility.

The ability of the ERs to flexibly bind to various ligands can be attributed to a number of unique features, as displayed in both ligand association as well as the binding cavity. In the classic example, E2 binding to ERs requires little contact between the receptor and the ligand in that only a portion of the molecule tightly fits within the binding pocket (Figure 8). The E2, as shown by X-ray crystal structures [32,34,63,64,78,81], fits the length and breadth of the pocket perfectly but leaves empty spaces (*i.e.*, sub-pockets) above and below the steroid core, particularly in the C and D ring regions [89]. The phenolic moiety (A ring) of the steroid core binds securely to the pocket by forming hydrophobic contacts with the residues situated above and below the ring, forming a network of hydrogen bonds with E353/305 and R394/346 (ERα and ERβ respectively) [5]. The C and D rings, on the other hand, are anchored to the pocket only by a single hydrogen bond between the D ring hydroxyl group and H524. Given the presence of sub-pockets, there exists adequate space to accommodate conformational changes [5] as well as a variety of substituents (e.g., 7α, 11β, 17α) with little to no steric clash in the vicinity [89].

Finally, the ER’s binding pockets have been shown to exhibit considerable structural plasticity. Larger ligands that would not have been expected to fit into the ER binding pocket have been reported to bind to the ER pocket as a result of the formation of novel binding grooves [30,55].

## 5. ER Specificity and Achieving Subtype Selectivity

Clearly, the ERs belie common lock and key dogma, instead showing considerable non-discriminatory nature in the ligands they will bind. ERs evolved this way to fulfill diverse and tissue-dependent biological roles, a characteristic highly conserved from ancestral receptor forms to present. The complexity of ER interaction grows further when subtype selectivity between the ERα and ERβ is considered.

In spite of the enormous structural diversity of compounds that bind to the ERs, the receptors exhibit high sensitivity to ligand structure as measured by differential binding affinity or downstream activation and signaling response. Minor modifications to ER-binding compounds will normally cause pronounced change in the binding affinity or even complete loss of activity [82,89]. For example, ketosteroids (e.g., androstenedione), although structurally similar to E2, do not bind to the ERs due to the switch from hydroxyl to ketone functional group in the A ring, which results in the disruption of the crucial hydrogen bond network.

Given that receptor promiscuity is by Nature’s design, it is not surprising that nature has also evolved alternate means to achieve specificity and, indeed, subtype selectivity. Some of the alternate ways of managing proper hormone signaling include elevating the concentration of circulating and tissue-specific hormone levels to the μM or mM range (e.g., 27OHC), and the use of “gating” enzymes (e.g., CYP7B1) to limit the access of certain hormones to a specific tissues [101]. Apart from these, the differences between the residues that lie within and beyond the binding pocket are widely known to play a major role in conferring ER subtype selectivity [106]. Nettles *et al.* [106] have used three compounds (THC, HPTE and PPT) to illustrate how the amino acid differences within and beyond the binding cavity can impact subtype selectivity as well as actions of these ligands. Impacts occur through the direct effects of different molecular interactions and through the indirect effects of long range interactions (arising from the differences in primary sequence beyond the binding pocket), both altering the selectivity of compounds. They have also identified structural differences in the surface, hydrophobic core, H12, β sheets and H1-H3 coils between the ERα and ERβ subtypes that caused differences in the cavity shape between the two ER subtypes (narrowing of back pocket of ERβ), which subsequently determine the different actions of ligands [106]. Indeed, unravelling the subtleties that lie between the ERα and ERβ has become the main impetus for many research efforts in the pursuit of designing more subtype selective ligands.

## 6. Updated Overview of Subtype Selective Compounds

A fair number of ERα and ERβ selective compounds and SERMs have been synthesized over the past decade, and a number of past reviews have provided summaries: a brief overview was provided by Redden [107] in 2004, followed by Zhao *et al.* [108], Henke and Heyer [109] and Veneeman [110] in 2005. Blizzard [111] in 2008 published a high level review of the SERMs investigated by Merck; and Minutolo *et al.* [112] provided an excellent review on ERβ ligands with specific focus on the ones published in 2005 to 2008.

Here a (non-exhaustive) summary that includes some of the most recent subtype selective ligands is provided. The intent is to delve into how subtype selectivity is achieved in these compounds, with particular focus given to how selectivity is mainly determined, as opposed to a full discussion on how the desired activity/therapeutic profile is attained.

Like the great majority of the ER subtype selective compounds, the selectivity of ERα and ERβ selective compounds discussed in the following sections, mostly arises from the interactions between certain moiety/ies in the small molecules with the two critical amino acids in the ERα and ERβ.

### 6.1. Updated Overview of ERα Selective Compounds

Table 3 lists the ERα selective ligands which will be reviewed in the section.

**Table 3:** ERα selective compounds with their respective fold selectivity for ERα. * Data for assays performed: the first number indicates data for ERα and the second number indicates data for ERβ; In superscript, ^B^ indicates binding assays, ^R^ indicates HEK 293 reporter gene assays. NA indicates that binding data was not reported. ID indicates the compound number that is cited in the text.

ID	Structure	Ref.	Fold Selectivity	Data *
1	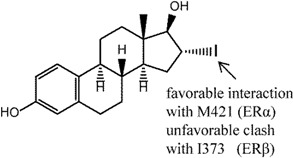	[113]	20–30 ^B^	NA
2	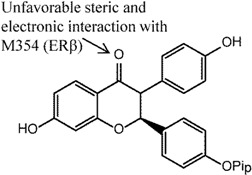	[114]	66 ^B^	(31 nM/2049 nM) ^B^
3	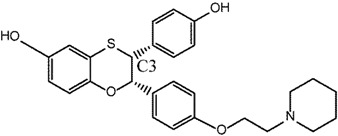	[115]	46 ^B^ 5.4 ^R^	(3.1 ± 1.4 nM/143 ± 72 nM)^B^ (9.6 nM/52 nM) ^R^
4	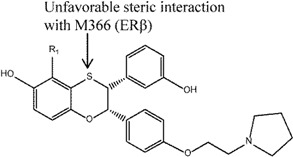	[116]	40 ^B^ 23.6 ^R^	(0.9 nM/37 nM) ^B^ (1.7 nM/40.1 nM) ^R^
5	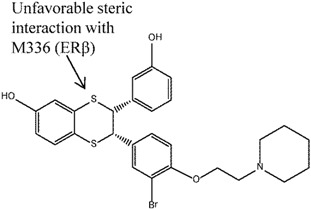	[117]	40 ^B^	(4 nM/161 nM) ^B^
6	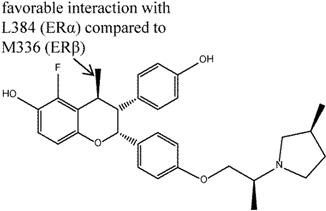	[44]	29 ^B^	(0.9 nM/26 nM) ^B^
7	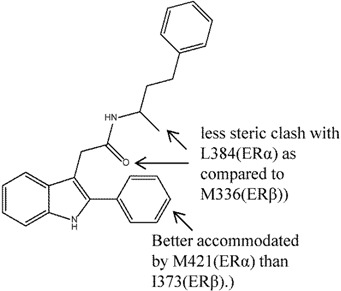	[51]	445 ^B^	(11 nM/4900 nM) ^B^
8	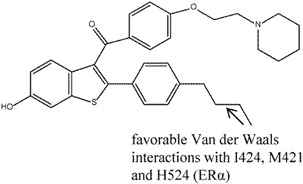	[118]	140 ^B^	(0.25 ± 0.15 nM/35 ± 14.3 nM) ^B^
9	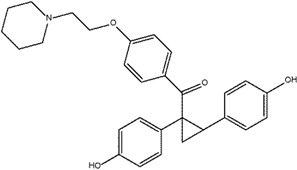	[119]	64 ^B^	(RBA: 0.64/0.01) ^B^

Compound **1** is 16α-iodo-17β estradiol (16αIE2) that represents a typical steroidal ER ligand. It has been shown to have 20-30 fold selectivity in binding affinity and 10-fold selectivity in activation towards the ERα subtype [113,120,121]. Bhat *et al.* [113] explored and provided molecular-level rationalization for the selectivity difference by using the ERα and ERβ chimeric receptors, four ER mutants (ERα L384M, ERα M421I, ERα L384M and M421I double mutant, ERβ I373M) as well as *in silico* studies. Specifically, they concluded that selectivity was stemmed from the LBD instead of from the rest of the receptor through ER chimera studies. Competitive ligand binding was used to study the binding affinities of 16αIE2 and E2 in ERα, ERβ and mutant of ERα receptors. The results showed that, while similar binding affinities were observed in the ERα LBD, a reduction in binding was observed for 16αIE2 compared to E2 in the ERβ and ERα mutant M421I, suggesting that the selectivity of 16αIE2 arose mainly from the favorable interactions with this M421 in ERα. Transactivation function assays were also performed on the wild-type ERs and their mutants using HepG2 cells and a 2X ERE-tk-luciferase reporter. Again, a difference in activation between 16αIE2 and E2 was observed in ERβ, ERα M421I and ERα L384M and M421I double mutant, which behaved similarly as ERα M421I, re-emphasizing the difference of M421 (ERα) and I373 (ERβ) as the main selectivity determinants for 16αIE2. Docking studies show that iodine substitution at the 16α position was found to cause two effects: (1) the iodine atom in 16αIE2 formed an unfavorable steric interaction with I373 of ERβ leading to a shift in 16αIE2 binding in the pocket relative to E2, which, in turn, increased the hydrogen bond distance of 17β-OH and H475 (3.9Å as compared to 2.9Å in E2-ERβ); and, (2) the iodine atom formed favorable interactions with M421 in ERα according to a quantum mechanics calculation [122].

Compounds **2** (a flavanone), **3** and **4** (dihydrobenzoxathiins), **5** (a dihydrobenzodithiin) and **6** (a chromane) comprise a group of closely related compounds derived from a series of structure activity relationship (SAR) efforts to design ERα selective SERMs (a.k.a SERAMs). These studies aimed to develop SERAMs that inhibit *in vitro* MCF-7 breast carcinoma cell growth and *in vivo* rat uterine weight gain

Compound **2** in the series of *cis*-2,3-disubstituted flavanones, with a 4-hydroxyphenyl substitution, was found to be most selective for ERα (66-fold) in ER competitive binding assay [114]. Selectivity for ERα was found to stem from the carbonyl group present in the ring, since its replacement with oxime or reduction to alcohol was found to decrease both binding and ERα selectivity. Molecular modeling showed that the crucial carbonyl had unfavorable steric and electronic interactions with M354 (note: different numbering, equivalent to M336 in this article) in ERβ, but not with ERα that had L384 in the corresponding position. An *in vivo* immature rat uterine weight assay was also performed to assess the agonist and antagonist activities of the compound wherein 50% inhibition was observed.

The dihydrobenzoxathiins, *i.e.*, compounds **3** and **4**, that exhibited significant ERα selectivity were extensively explored [115,116]. Similar to the flavanones, the unfavorable steric clash between the sulphur atom and M336 (ERβ) was suggested as the reason for the ERα selectivity [116]. In the preliminary study, Chen *et al.* [115] investigated substituents at the C3 position (compound **3**) and found that 4-hydroxyphenyl as preferable to alkyl, cycloalkyl and heteroaryl for ERα selectivity (46 fold selectivity for ERα in competitive binding assay). Removal of the hydroxyl group of 4-hydroxyphenyl was found to be detrimental to the selectivity. Cellular transactivation assay using HEK 293 cells stably co-transfected with either human ERα or ERβ was performed and showed selectivity towards ERα over ERβ (IC_50_ 9.6 nM and 52 nM, respectively). On the other hand, *in vivo* immature rat uterine assay demonstrated an inhibitory action of this compound (77% compared to 5% control). Kim *et al.* [116] investigated the effects of different substituents on the dihydrobenzoxathiins rings and found that, specifically at the R1 position in compound **4**, different substituents impacted both the binding affinity and ERα selectivity in both binding and cellular transactivation assays. In particular, any alkyl groups larger than methyl as well as electronegative substituents at this position led to reduced ERα selectivity. The former was found to not only reduce binding affinity for both ER subtypes but also reduced selectivity over ERβ. The latter (e.g., F, Cl substitutions) was suggested to cause reduced electronegativity of the adjacent S atom, leading to reduced electronic repulsion with M336 of ERβ, and subsequently increased ERβ binding affinity. Compound **4** was also found to be a potent inhibitor in an immature rat uterine weight gain assay. Modifications performed to the basic side chain of the dihyrobenzoxathiins were not found to affect the ERα selectivity [123].

Tan *et al.* [117] expanded the study of dihydrobenzoxathiins to dihydrobenzodithiin (compound **5**) and found that these compounds maintained the ERα selectivity property in binding assays, although antagonism was not observed in the rat uterine growth model (IC_50_ 152 nM in MCF-7 inhibition assay). Like the dihydrobenzoxathiins, the S atom was again found to be the key contributor to ERα selectivity of dihydrobenzodithiins.

In the same vein, the chromanes (e.g., compound **6**) were also explored and found to show ERα selective binding affinity [44], with selectivity attributable to the c4-trans methyl substitution to the *cis*-2,3-diphenylchromane structure. The transmethyl moiety, like the S atom in dihydrobenzoxathiins, confers the observed ERα selectivity. Study of the crystal structures of similar compounds suggested that this arose through left-shifted binding of these molecules in relation to the dihydrobezoxathiins, which led to closer interactions with L384 (ERα) but unfavorable interactions with M336 (ERβ). Compound **6** was also found to be a potent inhibitor in MCF-7 and immature rat uterine assays, and reduced serum cholesterol level to a greater extent than raloxifene.

Another group of compounds, the aryl-indoles, were also explored as potential SERAMs [51]. The acetamide linked 2-arylindole, compound **7**, was found to have up to 400-fold ERα selectivity in ER binding assay (although antagonism was observed in neither the rat uterine weight gain nor the MCF-7 cell proliferation assays). Again, the crystal structures reported in this study suggested that the selectivity was attributable to the difference in L384(ERα)/M336(ERβ) as well as M421(ERα)/I373(ERβ). The carbonyl moiety of acetamide and the methyl substituent of compound **7** experienced less steric clash with L384(ERα) as compared to M336(ERβ); the 2-phenyl substituent on the other hand, was better accommodated by M421(ERα) than I373(ERβ) [51].

Chalmers *et al.* [118] discovered a benzothiophene SERAM (BTPα) analogue, compound **8**, that had 140-fold ERα selectivity over ERβ (0.25 nM in ERα *vs.* 35 nM in ERβ in competitive binding assays). This compound was also found to possess good oral exposure after administration as well as antagonistic actions in MCF-7 assay (IC_50_ 33 nM). The crystal structure of ERα in complex with compound **8** revealed the binding mode of this compound in the binding pocket: the 6-OH group demonstrated similar binding mode as the equivalent in raloxifene, while the *n*-butyl group displaced the key H254 residue which was important for hydrogen bonding for raloxifene. The difference in selectivity of compound **8** between the two ER subtypes could not be identified due to the difficulty in obtaining an ERβ crystal structure in complex with compound **8**. On the other hand, hydrogen-deuterium exchange analysis showed that the biggest difference between ERα- and ER-β bound compound **8** was in the lower end of helix H7, where it appeared more stabilized in the former compared to the latter due to the increased contact and favorable Van der Waals interactions (ERα I424, M421 and H524) with the *n*-butyl moiety of compound **8** in ERα. Once again, the difference between one of the critical amino acids, M421in ERα *vs.* I373 in ERβ, was suggested to be the reason for the difference in selectivity; the favorable Van der Waals contact was disrupted by the branching of I373.

Yeo *et al.* [119] investigated the effects of replacing the double bonds of the stilbene structure with a cyclopropyl moiety (compound **9**) and discovered that this led to analogs with increased ERα selectivity, albeit with reduced binding affinity compared to E2 (measured through RBA). The cyclopropyl replacement led to the loss of planarity which resulted in different spatial arrangements that allowed the analogs to better distinguish the differences between the ERα and ERβ pockets (*i.e.*, increased selectivity) [119,124]. It appeared that the 4-OH substituted benzene ring gave the highest selectivity, and replacement of the hydroxyl group with methoxy led to decreased selectivity and binding affinity. The removal of the bulky basic side chain was found to increase the binding affinity of both subtypes, although selectivity was reduced. Compound **9** has also been found to demonstrate full agonist activity, however, a receptor-mediated gene transcription assay failed to show selectivity for compound **9** [119].

### 6.2. Updated Overview of ERβ Selective Compounds

Table 4 gives the ERβ selective ligands which will be reviewed in this section.

**Table 4:** ERβ selective compounds with their respective fold selectivity for ERβ. * Data for assays performed: the first number indicates data for ERβ and the second number indicates data for ERα; In superscript, ^B^ indicates binding assays, ^R^ indicates HEK 293 reporter gene assays. NA indicates where binding data is not available but functional data discussed in main text. ID indicates the compound number that is cited in the text.

ID	Structure	Ref	Fold Selectivity	Data *
10	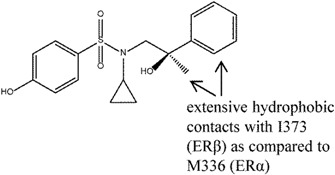	[80]	2.2 ^B^ 68.4 ^R^	(RBA: 107/48) ^B^ (>5400 nM/79 nM) ^R^
11	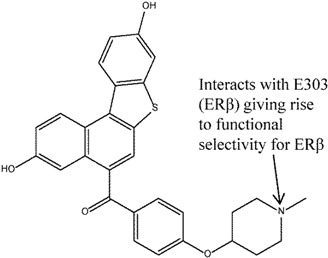	[125]	0.54 ^B^	(RBA: 0.038/0.07) ^B^
12	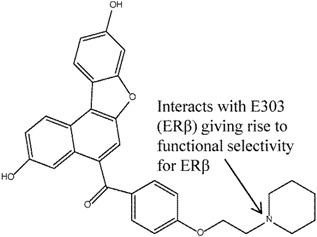	[125]	1.56 ^B^	(RBA:0.056/0.036) ^B^
13	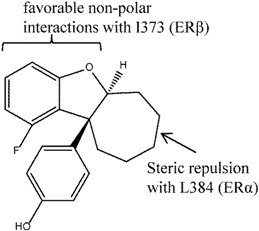	[124]	NA	NA
14	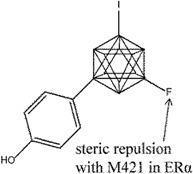	[126]	8.2 ^B^	(RBA: 61.1/7.8) ^B^
15	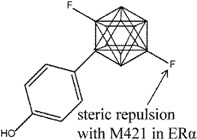	[126]	10.1 ^B^	(RBA: 49.9/4.9) ^B^
16	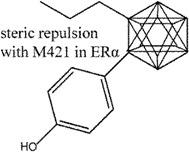	[127]	7.4 ^B^	(87/11.8) ^B^

**Figure 9:**
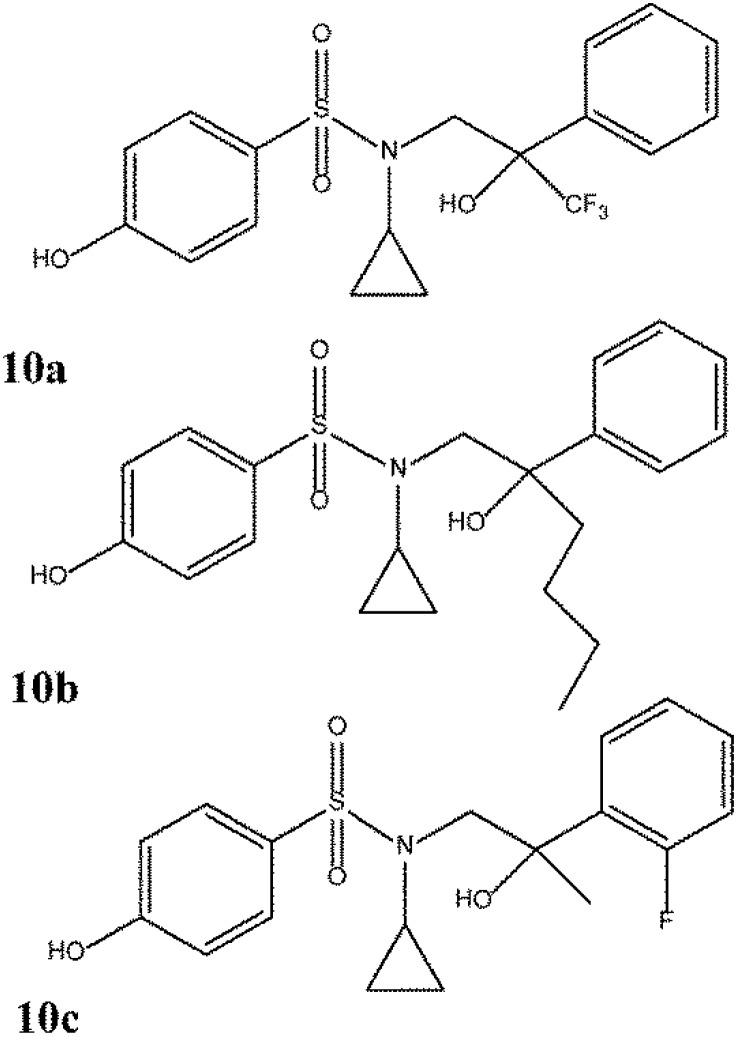
Analogues of ERβ selective compound **10**.

Roberts *et al.* [80] explored a series of *p*-hydroxybenzenesulphonamides as ERβ selective agonists. Compound **10** was identified as a compound of potential interest due to its satisfactory potency and efficacy in recombinant ligand binding domain and functional ERα and ERβ assays respectively. Study of the crystal structure of compound **10** bound to ERβ suggested that the selectivity arose from the extensive hydrophobic contacts with I373 (ERβ) as compared to M336 (ERα) with the benzyl and methyl moieties. Observations made from the X-ray structure prompted compound **10** to be used to produce compounds such as **10a**, **10b**, **10c** (Figure 9) with further improved potency and β-selectivity profile. The improvement in potency observed in compounds **10a**-**c** was believed to arise mainly from the additional substituents and subsequently increased lipophilicity and non-specific binding.

Rodriguez *et al.* [125] discovered full ERβ antagonists (compounds **11** and **12**) based on the benzonaphthofuran and benzonaphthothiophene skeletons. These compounds were interesting in the sense that, despite showing a lack of binding selectivity (relative binding affinity of compound **11**: ERα 0.07 *vs.* ERβ 0.038, compound **12**: ERα 0.036 *vs.* ERβ 0.056), they exhibited ERβ functional selectivity, *i.e.*, antagonistic activity at the μM range at ERβ but without detectable ERα activity at the same concentration range. Docking studies helped to rationalize the functional selectivity of these compounds for ERβ antagonistic activity. It appeared that, unlike the ER selective compounds mentioned earlier whose selectivity stemmed from the amino acid differences between ERα and ERβ, the selectivity determinant of these compounds seemed, rather, to be governed by their ability to form interactions between the basic side chains of the compounds with the critical E351/303 (ERα/ERβ). This crucial interaction, typical of SERMs, was found to be present in the majority of the docking solutions for ERβ with compounds **11** and **12**, but not for ERα. Furthermore, docking solutions were more easily generated for ERβ than for ERα.

**Figure 10:**
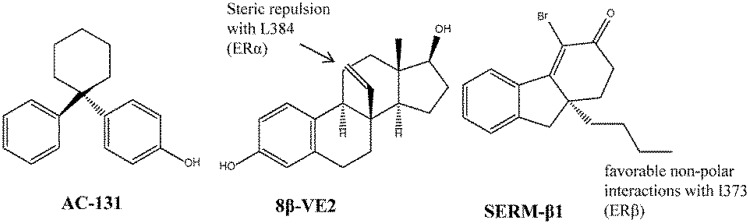
The non-steroidal AC-131 was used as the template in a SAR study to produce analogues such as compound **13** that showed ERβ selectivity. The feature that led to the ERβ selectivity for 8β-VE was repulsion from unfavorable interaction with M336 in ERβ, and for SERM-β1 was favorable hydrophobic contact with I373 in ERβ).

Sunden *et al.* [124] used compound AC-131 (Figure 10) as a template for enantio-selective SAR studies to generate the dihydrobenzofurans as ERβ agonists. The analogues generated were found to be highly potent (with EC_50_ as low as < 1 nM). While binding assays were not conducted, reporter gene assays showed 1,000-fold selectivity for ERβ over ERα with good potency of ~10 nM for compound **13** (*trans*-10-SS). A receptor selection and amplification technology (R-SAT) assay performed on this compound also demonstrated ERβ selectivity *i.e.*, pEC_50_ of 7.6 for ERβ and <5 for ERα. SAR indicated that a larger and flexible ring could potentially increase selectivity and activity. In particular, compound **13** with the larger cycloheptyl ring (as compared to cyclohexyl rings) achieved its remarkable ERβ selectivity in a manner similar to potent ERβ agonists, SERM-β1 [128] and 8β-VE2 (Figure 10) [129]. Docking and protein crystal structure studies of compound **10** and similar compounds, as well as SERM-β1, indicate that the benzofuran moiety faced I373 (as with the butyl group in SERM-β1), which led to favorable non-polar interactions, while the cycloheptyl group which was close to M336, led to steric repulsion (as with the vinyl group in 8β-VE2).

Ohta *et al.* explored carborane-containing compounds in terms of their ERβ specificity through substitution with fluorine [126] (compounds **14** and **15**) and aliphatic groups [127] (compound **16**). In the first study, fluorinated carboranyl phenol was investigated for ERβ selectivity, whereby the addition of a fluorine atom was found to increase the ERβ selectivity compared to non-fluorinated compounds in the series. The *m*-carboranyl phenol derivative (compound **14**) attained as high as 8.2 fold ERβ selectivity, while the *p*-carboranyl phenol derivative (compound **15**) reached as high as 10 fold ERβ selectivity based on a competitive ER binding assay. The selectivity of these compounds was suggested to result from repulsion with M421 of ERα (I373 in ERβ). Both compounds showed ER agonistic activities, albeit weaker than endogenous E2 [126]. Compounds **14** and **15** were also tested in MCF-7 assay where both compounds were found to promote cell proliferation in a dose-dependent manner, although as weaker agonists than E2.

In the second study [127], a series of carboranyl phenol derivatives with varying aliphatic substituents were investigated for binding affinity. Compound **16 ** was the most selective analogue in a competitive binding assay (7.4 fold selectivity for ERβ). This compound was also found to be a weaker agonist than E2 in a MCF-7 cell proliferation assay. Substitution performed on the carborane moiety in the *ortho* position was found to decrease the binding affinity of the analogues to both ERα and ERβ, but more so to ERα, thus increasing ERβ selectivity. It was found that substitution at the *meta* position did not produce ERβ selective compounds. Docking studies suggested that the selectivity of compound **16** may arise from the different binding modes of ERα and ERβ. For ERα, the *n*-butyl moiety of **16** found near the vicinity of M421 led to steric repulsion. For ERβ, the carborane instead of the *n*-butyl group was found in the same region, close to I373 (equivalent of M421 in ERα).

## 7. Conclusions

The ERs are an inherently versatile group of receptors, demonstrating a remarkable ability to recognize and interact with a wide range of small molecule ligands and consequently produce an impressive diversity of downstream responses. The disadvantage of this flexibility is manifested in an apparent non-discriminatory nature of the ERs in ligand binding that, unfortunately, often leads to a multitude of adverse health effects due to exogenous chemicals including those in the diet and environment. Though often co-expressed in certain tissue types, the ER subtypes are also differentially expressed in others [130]. In view of this, having ER subtype selective ligands is highly desirable to achieve selective and fine-tuned ER modulated responses in the course of disease treatments. A range of different subtype selective ER ligands have been discussed in this review. These selective ligands have been obtained through different strategies but mostly by the exploitation of the subtle differences in the ligand binding sites of ERα and ERβ. Typically, selectivity is achieved through the introduction of different substituents as well as through non-planarity to the small molecules – strategies that have been successful so far. All that said, the road to translate observed ER selective ligands to eventual clinical outcomes is not straightforward. It bears emphasizing that the lack of correlation between the binding affinity and efficacy should also be taken into consideration, at point illustrated by comparing E2 and genistein. While E2 binds with similar affinity to ERα and ERβ, it has been found to display higher transcriptional potency in ERα [106]. In contrast, despite binding with high affinity to the ERβ subtype (25 fold higher than ERα), genistein only acts as a partial agonist for the ERβ subtype [121]. Notwithstanding, the availability of more ER selective ligands will undeniably help to improve the clinical outcomes when treating ER mediated diseases by offering clinicians a wider selection of compounds to tailor to individual needs.

## References

[1] Paris M., Pettersson K., Schubert M., Bertrand S., Pongratz I., Escriva H., Laudet V. (2008). An amphioxus orthologue of the estrogen receptor that does not bind estradiol: Insights into estrogen receptor evolution. BMC Evol. Biol..

[2] Kampa M., Pelekanou V., Notas G., Stathopoulos E.N., Castanas E. (2013). The estrogen receptor: Two or more molecules, multiple variants, diverse localizations, signaling and functions. Are we undergoing a paradigm-shift as regards their significance in breast cancer?. Hormones (Athens).

[3] Beato M., Klug J. (2000). Steroid hormone receptors: An update. Hum. Reprod. Update.

[4] Gouva L., Tsatsoulis A. (2004). The role of estrogens in cardiovascular disease in the aftermath of clinical trials. Hormones (Athens).

[5] Ascenzi P., Bocedi A., Marino M. (2006). Structure-function relationship of estrogen receptor alpha and beta: Impact on human health. Mol. Aspects Med..

[6] Kavlock R.J., Daston G.P., DeRosa C., Fenner-Crisp P., Gray L.E., Kaattari S., Lucier G., Luster M., Mac M.J., Maczka C. (1996). Research needs for the risk assessment of health and environmental effects of endocrine disruptors: A report of the U.S. EPA-sponsored workshop. Environ. Health Perspect..

[7] Arnal J.F., Valera M.C., Payrastre B., Lenfant F., Gourdy P. (2012). Structure-function relationship of estrogen receptors in cardiovascular pathophysiological models. Thromb. Res..

[8] Lobo R.A. (2007). Menopause and stroke and the effects of hormonal therapy. Climacteric.

[9] Rossouw J.E., Anderson G.L., Prentice R.L., LaCroix A.Z., Kooperberg C., Stefanick M.L., Jackson R.D., Beresford S.A., Howard B.V., Johnson K.C. (2002). Risks and benefits of estrogen plus progestin in healthy postmenopausal women: Principal results from the women’s health initiative randomized controlled trial. JAMA.

[10] Muramatsu M., Inoue S. (2000). Estrogen receptors: How do they control reproductive and nonreproductive functions?. Biochem. Biophys. Res. Commun..

[11] Kuiper G.G., Enmark E., Pelto-Huikko M., Nilsson S., Gustafsson J.A. (1996). Cloning of a novel receptor expressed in rat prostate and ovary. Proc. Natl. Acad. Sci. USA.

[12] Hawkins M.B., Thornton J.W., Crews D., Skipper J.K., Dotte A., Thomas P. (2000). Identification of a third distinct estrogen receptor and reclassification of estrogen receptors in teleosts. Proc. Natl. Acad. Sci. USA.

[13] Xu X., Yang W., Li Y., Wang Y. (2010). Discovery of estrogen receptor modulators: A review of virtual screening and SAR efforts. Expert Opin. Drug Discov..

[14] Koehler K.F., Helguero L.A., Haldosen L.A., Warner M., Gustafsson J.A. (2005). Reflections on the discovery and significance of estrogen receptor beta. Endocr. Rev..

[15] Paruthiyil S., Parmar H., Kerekatte V., Cunha G.R., Firestone G.L., Leitman D.C. (2004). Estrogen receptor beta inhibits human breast cancer cell proliferation and tumor formation by causing a G2 cell cycle arrest. Cancer Res..

[16] Maggiolini M., Bonofiglio D., Marsico S., Panno M.L., Cenni B., Picard D., Ando S. (2001). Estrogen receptor alpha mediates the proliferative but not the cytotoxic dose-dependent effects of two major phytoestrogens on human breast cancer cells. Mol. Pharmacol..

[17] Rizza P., Barone I., Zito D., Giordano F., Lanzino M., De Amicis F., Mauro L., Sisci D., Catalano S., Wright K.D. (2014). Estrogen receptor beta as a novel target of androgen receptor action in breast cancer cell lines. Breast Cancer Res..

[18] Ruff M., Gangloff M., Marie Wurtz J., Moras D. (2000). Estrogen receptor transcription and transactivation: Structure-function relationship in DNA- and ligand-binding domains of estrogen receptors. Breast Cancer Res..

[19] Germain P., Staels B., Dacquet C., Spedding M., Laudet V. (2006). Overview of nomenclature of nuclear receptors. Pharmacol. Rev..

[20] Benoit G., Cooney A., Giguere V., Ingraham H., Lazar M., Muscat G., Perlmann T., Renaud J.P., Schwabe J., Sladek F. (2006). International Union of Pharmacology. LXVI. Orphan nuclear receptors. Pharmacol. Rev..

[21] Gearhart M.D., Holmbeck S.M., Evans R.M., Dyson H.J., Wright P.E. (2003). Monomeric complex of human orphan estrogen related receptor-2 with DNA: A pseudo-dimer interface mediates extended half-site recognition. J. Mol. Biol..

[22] Forman B.M., Samuels H.H. (1990). Dimerization among nuclear hormone receptors. New. Biol..

[23] Sotoca A.M., Vervoort J., Rietjens I.M.C.M., Gustafsson J. (2012). Human ERα and ERβ Splice Variants: Understanding Their Domain Structure in Relation to Their Biological Roles in Breast Cancer Cell Proliferation.

[24] Poola I., Koduri S., Chatra S., Clarke R. (2000). Identification of twenty alternatively spliced estrogen receptor alpha mRNAs in breast cancer cell lines and tumors using splice targeted primer approach. J. Steroid Biochem. Mol. Boil..

[25] Lewandowski S., Kalita K., Kaczmarek L. (2002). Estrogen receptor beta. Potential functional significance of a variety of mRNA isoforms. FEBS Lett..

[26] Figtree G.A., McDonald D., Watkins H., Channon K.M. (2003). Truncated estrogen receptor alpha 46-kDa isoform in human endothelial cells: Relationship to acute activation of nitric oxide synthase. Circulation.

[27] Leung Y.K., Mak P., Hassan S., Ho S.M. (2006). Estrogen receptor (ER)-beta isoforms: A key to understanding ER-beta signaling. Proc. Natl. Acad. Sci. USA.

[28] Pink J.J., Wu S.Q., Wolf D.M., Bilimoria M.M., Jordan V.C. (1996). A novel 80 kDa human estrogen receptor containing a duplication of exons 6 and 7. Nucl. Acids Res..

[29] Tanenbaum D.M., Wang Y., Williams S.P., Sigler P.B. (1998). Crystallographic comparison of the estrogen and progesterone receptor’s ligand binding domains. Proc. Natl. Acad. Sci. USA.

[30] Brzozowski A.M., Pike A.C., Dauter Z., Hubbard R.E., Bonn T., Engstrom O., Ohman L., Greene G.L., Gustafsson J.A., Carlquist M. (1997). Molecular basis of agonism and antagonism in the oestrogen receptor. Nature.

[31] Shiau A.K., Barstad D., Loria P.M., Cheng L., Kushner P.J., Agard D.A., Greene G.L. (1998). The structural basis of estrogen receptor/coactivator recognition and the antagonism of this interaction by tamoxifen. Cell.

[32] Gangloff M., Ruff M., Eiler S., Duclaud S., Wurtz J.M., Moras D. (2001). Crystal structure of a mutant hERalpha ligand-binding domain reveals key structural features for the mechanism of partial agonism. J. Biol. Chem..

[33] Eiler S., Gangloff M., Duclaud S., Moras D., Ruff M. (2001). Overexpression, purification, and crystal structure of native ER alpha LBD. Protein Exp. Purif..

[34] Warnmark A., Treuter E., Gustafsson J.A., Hubbard R.E., Brzozowski A.M., Pike A.C. (2002). Interaction of transcriptional intermediary factor 2 nuclear receptor box peptides with the coactivator binding site of estrogen receptor alpha. J. Biol. Chem..

[35] Shiau A.K., Barstad D., Radek J.T., Meyers M.J., Nettles K.W., Katzenellenbogen B.S., Katzenellenbogen J.A., Agard D.A., Greene G.L. (2002). Structural characterization of a subtype-selective ligand reveals a novel mode of estrogen receptor antagonism. Nat. Struct. Biol..

[36] Leduc A.M., Trent J.O., Wittliff J.L., Bramlett K.S., Briggs S.L., Chirgadze N.Y., Wang Y., Burris T.P., Spatola A.F. (2003). Helix-stabilized cyclic peptides as selective inhibitors of steroid receptor-coactivator interactions. Proc. Natl. Acad. Sci. USA.

[37] Renaud J., Bischoff S.F., Buhl T., Floersheim P., Fournier B., Halleux C., Kallen J., Keller H., Schlaeppi J.M., Stark W. (2003). Estrogen receptor modulators: Identification and structure-activity relationships of potent ERalpha-selective tetrahydroisoquinoline ligands. J. Med. Chem..

[38] Wu Y.L., Yang X., Ren Z., McDonnell D.P., Norris J.D., Willson T.M., Greene G.L. (2005). Structural basis for an unexpected mode of serm-mediated ER antagonism. Mol. Cell.

[39] Kim S., Wu J.Y., Birzin E.T., Frisch K., Chan W., Pai L.Y., Yang Y.T., Mosley R.T., Fitzgerald P.M., Sharma N. (2004). Estrogen receptor ligands. II. Discovery of benzoxathiins as potent, selective estrogen receptor alpha modulators. J. Med. Chem..

[40] Blizzard T.A., Dininno F., Morgan J.D., Chen H.Y., Wu J.Y., Kim S., Chan W., Birzin E.T., Yang Y.T., Pai L.Y. (2005). Estrogen receptor ligands. Part 9: Dihydrobenzoxathiin serams with alkyl substituted pyrrolidine side chains and linkers. Bioorg. Med. Chem. Lett..

[41] Manas E.S., Unwalla R.J., Xu Z.B., Malamas M.S., Miller C.P., Harris H.A., Hsiao C., Akopian T., Hum W.T., Malakian K. (2004). Structure-based design of estrogen receptor-beta selective ligands. J. Am. Chem. Soc..

[42] Manas E.S., Xu Z.B., Unwalla R.J., Somers W.S. (2004). Understanding the selectivity of genistein for human estrogen receptor-beta using X-ray crystallography and computational methods. Structure.

[43] Renaud J., Bischoff S.F., Buhl T., Floersheim P., Fournier B., Geiser M., Halleux C., Kallen J., Keller H., Ramage P. (2005). Selective estrogen receptor modulators with conformationally restricted side chains. Synthesis and structure-activity relationship of ERalpha-selective tetrahydroisoquinoline ligands. J. Med. Chem..

[44] Tan Q., Blizzard T.A., Morgan J.D., Birzin E.T., Chan W., Yang Y.T., Pai L.Y., Hayes E.C., DaSilva C.A., Warrier S. (2005). Estrogen receptor ligands. Part 10: Chromanes: Old scaffolds for new SERAMs. Bioorg. Med. Chem. Lett..

[45] Hummel C.W., Geiser A.G., Bryant H.U., Cohen I.R., Dally R.D., Fong K.C., Frank S.A., Hinklin R., Jones S.A., Lewis G. (2005). A selective estrogen receptor modulator designed for the treatment of uterine leiomyoma with unique tissue specificity for uterus and ovaries in rats. J. Med. Chem..

[46] Nettles K.W., Bruning J.B., Gil G., Nowak J., Sharma S.K., Hahm J.B., Kulp K., Hochberg R.B., Zhou H., Katzenellenbogen J.A. (2008). Nfkappab selectivity of estrogen receptor ligands revealed by comparative crystallographic analyses. Nat. Chem. Biol..

[47] Kong E.H., Heldring N., Gustafsson J.A., Treuter E., Hubbard R.E., Pike A.C. (2005). Delineation of a unique protein-protein interaction site on the surface of the estrogen receptor. Proc. Natl. Acad. Sci. USA.

[48] Hsieh R.W., Rajan S.S., Sharma S.K., Guo Y., DeSombre E.R., Mrksich M., Greene G.L. (2006). Identification of ligands with bicyclic scaffolds provides insights into mechanisms of estrogen receptor subtype selectivity. J. Biol. Chem..

[49] Hsieh R.W., Rajan S.S., Sharma S.K., Greene G.L. (2008). Molecular characterization of a B-ring unsaturated estrogen: Implications for conjugated equine estrogen components of premarin. Steroids.

[50] Norman B.H., Richardson T.I., Dodge J.A., Pfeifer L.A., Durst G.L., Wang Y., Durbin J.D., Krishnan V., Dinn S.R., Liu S. (2007). Benzopyrans as selective estrogen receptor beta agonists (SERBAs). Part 4: Functionalization of the benzopyran A-ring. Bioorg. Med. Chem. Lett..

[51] Dykstra K.D., Guo L., Birzin E.T., Chan W., Yang Y.T., Hayes E.C., DaSilva C.A., Pai L.Y., Mosley R.T., Kraker B. (2007). Estrogen receptor ligands. Part 16: 2-aryl indoles as highly subtype selective ligands for ERalpha. Bioorg. Med. Chem. Lett..

[52] Heldring N., Pawson T., McDonnell D., Treuter E., Gustafsson J.A., Pike A.C. (2007). Structural insights into corepressor recognition by antagonist-bound estrogen receptors. J. Biol. Chem..

[53] Koide A., Abbatiello S., Rothgery L., Koide S. (2002). Probing protein conformational changes in living cells by using designer binding proteins: Application to the estrogen receptor. Proc. Natl. Acad. Sci. USA.

[54] Vajdos F.F., Hoth L.R., Geoghegan K.F., Simons S.P., LeMotte P.K., Danley D.E., Ammirati M.J., Pandit J. (2007). The 2.0 a crystal structure of the ERalpha ligand-binding domain complexed with lasofoxifene. Protein Sci..

[55] Nettles K.W., Bruning J.B., Gil G., O’Neill E.E., Nowak J., Guo Y., Kim Y., DeSombre E.R., Dilis R., Hanson R.N. (2007). Structural plasticity in the oestrogen receptor ligand-binding domain. EMBO Rep..

[56] Richardson T.I., Norman B.H., Lugar C.W., Jones S.A., Wang Y., Durbin J.D., Krishnan V., Dodge J.A. (2007). Benzopyrans as selective estrogen receptor beta agonists (SERBAs). Part 2: Structure-activity relationship studies on the benzopyran scaffold. Bioorg. Med. Chem. Lett..

[57] Zhou H.B., Nettles K.W., Bruning J.B., Kim Y., Joachimiak A., Sharma S., Carlson K.E., Stossi F., Katzenellenbogen B.S., Greene G.L. (2007). Elemental isomerism: A boron-nitrogen surrogate for a carbon-carbon double bond increases the chemical diversity of estrogen receptor ligands. Chem. Biol..

[58] Richardson T.I., Dodge J.A., Durst G.L., Pfeifer L.A., Shah J., Wang Y., Durbin J.D., Krishnan V., Norman B.H. (2007). Benzopyrans as selective estrogen receptor beta agonists (SERBAs). Part 3: Synthesis of cyclopentanone and cyclohexanone intermediates for C-ring modification. Bioorg. Med. Chem. Lett..

[59] Bruning J.B., Parent A.A., Gil G., Zhao M., Nowak J., Pace M.C., Smith C.L., Afonine P.V., Adams P.D., Katzenellenbogen J.A. (2010). Coupling of receptor conformation and ligand orientation determine graded activity. Nat. Chem. Biol..

[60] Dai S.Y., Chalmers M.J., Bruning J., Bramlett K.S., Osborne H.E., Montrose-Rafizadeh C., Barr R.J., Wang Y., Wang M., Burris T.P. (2008). Prediction of the tissue-specificity of selective estrogen receptor modulators by using a single biochemical method. Proc. Natl. Acad. Sci. USA.

[61] Fang J., Akwabi-Ameyaw A., Britton J.E., Katamreddy S.R., Navas F., Miller A.B., Williams S.P., Gray D.W., Orband-Miller L.A., Shearin J. (2008). Synthesis of 3-alkyl naphthalenes as novel estrogen receptor ligands. Bioorg. Med. Chem. Lett..

[62] Li M.J., Greenblatt H.M., Dym O., Albeck S., Pais A., Gunanathan C., Milstein D., Degani H., Sussman J.L. (2011). Structure of estradiol metal chelate and estrogen receptor complex: The basis for designing a new class of selective estrogen receptor modulators. J. Med. Chem..

[63] Phillips C., Roberts L.R., Schade M., Bazin R., Bent A., Davies N.L., Moore R., Pannifer A.D., Pickford A.R., Prior S.H. (2011). Design and structure of stapled peptides binding to estrogen receptors. J. Am. Chem. Soc..

[64] Delfosse V., Grimaldi M., Pons J.L., Boulahtouf A., le Maire A., Cavailles V., Labesse G., Bourguet W., Balaguer P. (2012). Structural and mechanistic insights into bisphenols action provide guidelines for risk assessment and discovery of bisphenol a substitutes. Proc. Natl. Acad. Sci. USA.

[65] Osz J., Brelivet Y., Peluso-Iltis C., Cura V., Eiler S., Ruff M., Bourguet W., Rochel N., Moras D. (2012). Structural basis for a molecular allosteric control mechanism of cofactor binding to nuclear receptors. Proc. Natl. Acad. Sci. USA.

[66] Srinivasan S., Nwachukwu J.C., Parent A.A., Cavett V., Nowak J., Hughes T.S., Kojetin D.J., Katzenellenbogen J.A., Nettles K.W. (2013). Ligand-binding dynamics rewire cellular signaling via estrogen receptor-alpha. Nat. Chem. Biol..

[67] Pike A.C., Brzozowski A.M., Hubbard R.E., Bonn T., Thorsell A.G., Engstrom O., Ljunggren J., Gustafsson J.A., Carlquist M. (1999). Structure of the ligand-binding domain of oestrogen receptor beta in the presence of a partial agonist and a full antagonist. Embo J..

[68] Pike A.C., Brzozowski A.M., Walton J., Hubbard R.E., Thorsell A.G., Li Y.L., Gustafsson J.A., Carlquist M. (2001). Structural insights into the mode of action of a pure antiestrogen. Structure.

[69] Henke B.R., Consler T.G., Go N., Hale R.L., Hohman D.R., Jones S.A., Lu A.T., Moore L.B., Moore J.T., Orband-Miller L.A. (2002). A new series of estrogen receptor modulators that display selectivity for estrogen receptor beta. J. Med. Chem..

[70] Malamas M.S., Manas E.S., McDevitt R.E., Gunawan I., Xu Z.B., Collini M.D., Miller C.P., Dinh T., Henderson R.A., Keith J.C. (2004). Design and synthesis of aryl diphenolic azoles as potent and selective estrogen receptor-beta ligands. J. Med. Chem..

[71] Mewshaw R.E., Edsall R.J., Yang C., Manas E.S., Xu Z.B., Henderson R.A., Keith J.C., Harris H.A. (2005). Erbeta ligands. 3. Exploiting two binding orientations of the 2-phenylnaphthalene scaffold to achieve ERbeta selectivity. J. Med. Chem..

[72] McDevitt R.E., Malamas M.S., Manas E.S., Unwalla R.J., Xu Z.B., Miller C.P., Harris H.A. (2005). Estrogen receptor ligands: Design and synthesis of new 2-arylindene-1-ones. Bioorg. Med. Chem. Lett..

[73] Wang Y., Chirgadze N.Y., Briggs S.L., Khan S., Jensen E.V., Burris T.P. (2006). A second binding site for hydroxytamoxifen within the coactivator-binding groove of estrogen receptor beta. Proc. Natl. Acad. Sci. USA.

[74] Wilkening R.R., Ratcliffe R.W., Tynebor E.C., Wildonger K.J., Fried A.K., Hammond M.L., Mosley R.T., Fitzgerald P.M., Sharma N., McKeever B.M. (2006). The discovery of tetrahydrofluorenones as a new class of estrogen receptor beta-subtype selective ligands. Bioorg. Med. Chem. Lett..

[75] Norman B.H., Dodge J.A., Richardson T.I., Borromeo P.S., Lugar C.W., Jones S.A., Chen K., Wang Y., Durst G.L., Barr R.J. (2006). Benzopyrans are selective estrogen receptor beta agonists with novel activity in models of benign prostatic hyperplasia. J. Med. Chem..

[76] Mewshaw R.E., Bowen S.M., Harris H.A., Xu Z.B., Manas E.S., Cohn S.T. (2007). ERbeta ligands. Part 5: Synthesis and structure-activity relationships of a series of 4’-hydroxyphenyl-aryl-carbaldehyde oxime derivatives. Bioorg. Med. Chem. Lett..

[77] Richardson T.I., Dodge J.A., Wang Y., Durbin J.D., Krishnan V., Norman B.H. (2007). Benzopyrans as selective estrogen receptor beta agonists (SERBAs). Part 5: Combined A- and C-ring structure-activity relationship studies. Bioorg. Med. Chem. Lett..

[78] Mocklinghoff S., Rose R., Carraz M., Visser A., Ottmann C., Brunsveld L. (2010). Synthesis and crystal structure of a phosphorylated estrogen receptor ligand binding domain. Chembiochem.

[79] Mocklinghoff S., van Otterlo W.A., Rose R., Fuchs S., Zimmermann T.J., Dominguez Seoane M., Waldmann H., Ottmann C., Brunsveld L. (2011). Design and evaluation of fragment-like estrogen receptor tetrahydroisoquinoline ligands from a scaffold-detection approach. J. Med. Chem..

[80] Roberts L.R., Armor D., Barker C., Bent A., Bess K., Brown A., Favor D.A., Ellis D., Irving S.L., MacKenny M. (2011). Sulfonamides as selective oestrogen receptor beta agonists. Bioorg. Med. Chem. Lett..

[81] Fuchs S., Nguyen H.D., Phan T.T., Burton M.F., Nieto L., de Vries-van Leeuwen I.J., Schmidt A., Goodarzifard M., Agten S.M., Rose R. (2013). Proline primed helix length as a modulator of the nuclear receptor-coactivator interaction. J. Am. Chem. Soc..

[82] Pike A.C. (2006). Lessons learnt from structural studies of the oestrogen receptor. Best Pract. Res. Clin. Endocrinol. Metab..

[83] Hickey M., Hart R., Keelan J.A. (2014). The relationship between umbilical cord estrogens and perinatal characteristics: Implications for early life origins of reproductive cancers. Cancer Epidemiol. Biomarkers Prev..

[84] Ojasoo T., Raynaud J.P., Dore J.C. (1995). Correspondence factor analysis of steroid libraries. Steroids.

[85] Ding D., Xu L., Fang H., Hong H., Perkins R., Harris S., Bearden E.D., Shi L., Tong W. (2010). The EDKB: An established knowledge base for endocrine disrupting chemicals. BMC Bioinformatics.

[86] Shen J., Xu L., Fang H., Richard A.M., Bray J.D., Judson R.S., Zhou G., Colatsky T.J., Aungst J.L., Teng C. (2013). EADB: An estrogenic activity database for assessing potential endocrine activity. Toxicol. Sci..

[87] Diamanti-Kandarakis E., Bourguignon J.P., Giudice L.C., Hauser R., Prins G.S., Soto A.M., Zoeller R.T., Gore A.C. (2009). Endocrine-disrupting chemicals: An endocrine society scientific statement. Endocr. Rev..

[88] Jiang Y., Gong P., Madak-Erdogan Z., Martin T., Jeyakumar M., Carlson K., Khan I., Smillie T.J., Chittiboyina A.G., Rotte S.C. (2013). Mechanisms enforcing the estrogen receptor beta selectivity of botanical estrogens. FASEB J..

[89] Anstead G.M., Carlson K.E., Katzenellenbogen J.A. (1997). The estradiol pharmacophore: Ligand structure-estrogen receptor binding affinity relationships and a model for the receptor binding site. Steroids.

[90] Delettre J., Mornon J.P., Lepicard G., Ojasoo T., Raynaud J.P. (1980). Steroid flexibility and receptor specificity. J. Steroid Biochem..

[91] Hong H., Tong W., Fang H., Shi L., Xie Q., Wu J., Perkins R., Walker J.D., Branham W., Sheehan D.M. (2002). Prediction of estrogen receptor binding for 58,000 chemicals using an integrated system of a tree-based model with structural alerts. Environ. Health Perspect..

[92] Shi L., Tong W., Fang H., Xie Q., Hong H., Perkins R., Wu J., Tu M., Blair R.M., Branham W.S. (2002). An integrated “4-phase” approach for setting endocrine disruption screening priorities—Phase I and II predictions of estrogen receptor binding affinity. SAR QSAR Environ. Res..

[93] Birnbaum L.S. (2013). State of the science of endocrine disruptors. Environ. Health Perspect..

[94] Malone K.E. (1993). Diethylstilbestrol (DES) and breast cancer. Epidemiol. Rev..

[95] Palmer J.R., Hatch E.E., Rosenberg C.L., Hartge P., Kaufman R.H., Titus-Ernstoff L., Noller K.L., Herbst A.L., Rao R.S., Troisi R. (2002). Risk of breast cancer in women exposed to diethylstilbestrol *in utero*: Preliminary results (United States). Cancer Causes Control.

[96] Noller K.L., Fish C.R. (1974). Diethylstilbestrol usage: Its interesting past, important present, and questionable future. Med. Clin. North Am..

[97] Piver M.S., Lele S.B., Baker T.R., Sandecki A. (1988). Cervical and vaginal cancer detection at a regional diethylstilbestrol (DES) screening clinic. Cancer Detect. Prev..

[98] Verloop J., van Leeuwen F.E., Helmerhorst T.J., van Boven H.H., Rookus M.A. (2010). Cancer risk in DES daughters. Cancer Causes Control.

[99] State of the Art Assessment of Endocrine Disrupters Final Report. http://ec.europa.eu/environment/chemicals/endocrine/pdf/sota_edc_final_report.pdf.

[100] Schug T.T., Janesick A., Blumberg B., Heindel J.J. (2011). Endocrine disrupting chemicals and disease susceptibility. J. Steroid Biochem. Mol. Biol..

[101] Lathe R., Kotelevtsev Y. (2014). Steroid signaling: Ligand-binding promiscuity, molecular symmetry, and the need for gating. Steroids.

[102] DuSell C.D., Umetani M., Shaul P.W., Mangelsdorf D.J., McDonnell D.P. (2008). 27-hydroxycholesterol is an endogenous selective estrogen receptor modulator. Mol. Endocrinol..

[103] Saijo K., Collier J.G., Li A.C., Katzenellenbogen J.A., Glass C.K. (2011). An adiol-ERbeta-CTBP transrepression pathway negatively regulates microglia-mediated inflammation. Cell.

[104] Umetani M., Shaul P.W. (2011). 27-hydroxycholesterol: The first identified endogenous SERM. Trends Endocrinol. Metab..

[105] Fleming F.J., Hill A.D.K., McDermott E.W., O’Higgins N.J., Young L.S. (2004). Differential recruitment of coregulator proteins steroid receptor coactivator-1 and silencing mediator for retinoid and thyroid receptors to the estrogen receptor-estrogen response element by β-estradiol and 4-hydroxytamoxifen in human breast cancer. J. Clin. Endocrinol. Metab..

[106] Nettles K.W., Sun J., Radek J.T., Sheng S., Rodriguez A.L., Katzenellenbogen J.A., Katzenellenbogen B.S., Greene G.L. (2004). Allosteric control of ligand selectivity between estrogen receptors alpha and beta: Implications for other nuclear receptors. Mol. Cell..

[107] Redden P.R. (2004). Selective oestrogen receptor modulators, pure antioestrogens and related oestrogen receptor ligands. Expert Opin. Ther. Patents.

[108] Zhao L., O’Neill K., Diaz Brinton R. (2005). Selective estrogen receptor modulators (SERMs) for the brain: Current status and remaining challenges for developing NeuroSERMs. Brain Res. Brain Res. Rev..

[109] Henke B.R., Heyer D. (2005). Recent advances in estrogen receptor modulators. Curr. Opin. Drug Discov. Devel..

[110] Veeneman G.H. (2005). Non-steroidal subtype selective estrogens. Curr. Med. Chem.

[111] Blizzard T.A. (2008). Selective estrogen receptor modulator medicinal chemistry at Merck. A review. Curr. Top. Med. Chem..

[112] Minutolo F., Macchia M., Katzenellenbogen B.S., Katzenellenbogen J.A. (2011). Estrogen receptor beta ligands: Recent advances and biomedical applications. Med. Res. Rev..

[113] Bhat R.A., Stauffer B., Unwalla R.J., Xu Z., Harris H.A., Komm B.S. (2004). Molecular determinants of ER alpha and ER beta involved in selectivity of 16 alpha-iodo-7 beta estradiol. J. Steroid Biochem. Mol. Biol..

[114] Chen H.Y., Dykstra K.D., Birzin E.T., Frisch K., Chan W., Yang Y.T., Mosley R.T., DiNinno F., Rohrer S.P., Schaeffer J.M. (2004). Estrogen receptor ligands. Part 1: The discovery of flavanoids with subtype selectivity. Bioorg. Med. Chem. Lett..

[115] Chen H.Y., Kim S., Wu J.Y., Birzin E.T., Chan W., Yang Y.T., Dahllund J., DiNinno F., Rohrer S.P., Schaeffer J.M. (2004). Estrogen receptor ligands. Part 3: The SAR of dihydrobenzoxathiin SERMs. Bioorg. Med. Chem. Lett..

[116] Kim S., Wu J., Chen H.Y., Birzin E.T., Chan W., Yang Y.T., Colwell L., Li S., Dahllund J., DiNinno F. (2004). Estrogen receptor ligands. Part 4: The SAR of the syn-dihydrobenzoxathiin SERAMs. Bioorg. Med. Chem. Lett..

[117] Tan Q., Birzin E.T., Chan W., Yang Y.T., Pai L.Y., Hayes E.C., DaSilva C.A., DiNinno F., Rohrer S.P., Schaeffer J.M. (2004). Estrogen receptor ligands. Part 6: Synthesis and binding affinity of dihydrobenzodithiins. Bioorg. Med. Chem. Lett..

[118] Chalmers M.J., Wang Y., Novick S., Sato M., Bryant H.U., Montrose-Rafizdeh C., Griffin P.R., Dodge J.A. (2012). Hydrophobic interactions improve selectivity to ERalpha for ben-zothiophene serms. ACS Med. Chem. Lett..

[119] Yeo H.L., Song Y.S., Ryu J.H., Kim H.D. (2013). Design, synthesis, and biological evaluation of cyclopropyl analogues of stilbene with raloxifene side chain as subtype-selective ligands for estrogen receptor. Arch. Pharm. Res..

[120] Kuiper G.G., Carlsson B., Grandien K., Enmark E., Haggblad J., Nilsson S., Gustafsson J.A. (1997). Comparison of the ligand binding specificity and transcript tissue distribution of estrogen receptors alpha and beta. Endocrinology.

[121] Kuiper G.G., Lemmen J.G., Carlsson B., Corton J.C., Safe S.H., van der Saag P.T., van der Burg B., Gustafsson J.A. (1998). Interaction of estrogenic chemicals and phytoestrogens with estrogen receptor beta. Endocrinology.

[122] Unwalla R.J., Manas E.S., Miller C.P., Xu Z. Computational approaches to understand selectivity between receptors alpha and beta. Proceedings of the 226 ACS National Meeting.

[123] Tan Q., Birzin E.T., Chan W., Tien Yang Y., Pai L.Y., Hayes E.C., DaSilva C.A., DiNinno F., Rohrer S.P., Schaeffer J.M. (2004). Estrogen receptor ligands. Part 5: The SAR of dihydrobenzoxathiins containing modified basic side chains. Bioorg. Med. Chem. Lett..

[124] Sunden H., Ma J.N., Hansen L.K., Gustavsson A.L., Burstein E.S., Olsson R. (2013). Design of a highly selective and potent class of non-planar estrogen receptor beta agonists. ChemMedChem.

[125] Rodriguez J.J., Filipiak K., Maslyk M., Ciepielski J., Demkowicz S., de Pascual-Teresa S., Martin-Santamaria S., de Pascual-Teresa B., Ramos A. (2012). Towards beta-selectivity in functional estrogen receptor antagonists. Org. Biomol. Chem..

[126] Ohta K., Ogawa T., Kaise A., Endo Y. (2013). Enhanced estrogen receptor beta (ERbeta) selectivity of fluorinated carborane-containing ER modulators. Bioorg. Med. Chem. Lett..

[127] Ohta K., Ogawa T., Kaise A., Oda A., Endo Y. (2014). Aliphatic substitution of o-carboranyl phenols enhances estrogen receptor beta selectivity. Chem. Pharm. Bull. (Tokyo).

[128] Wilkening R.R., Ratcliffe R.W., Fried A.K., Meng D., Sun W., Colwell L., Lambert S., Greenlee M., Nilsson S., Thorsell A. (2006). Estrogen receptor beta-subtype selective tetrahydrofluorenones: Use of a fused pyrazole as a phenol bioisostere. Bioorg. Med. Chem. Lett..

[129] Hegele-Hartung C., Siebel P., Peters O., Kosemund D., Muller G., Hillisch A., Walter A., Kraetzschmar J., Fritzemeier K.H. (2004). Impact of isotype-selective estrogen receptor agonists on ovarian function. Proc. Natl. Acad. Sci. USA.

[130] Harrington W.R., Sheng S., Barnett D.H., Petz L.N., Katzenellenbogen J.A., Katzenellenbogen B.S. (2003). Activities of estrogen receptor alpha- and beta-selective ligands at diverse estrogen responsive gene sites mediating transactivation or transrepression. Mol. Cell. Endocrinol..

